# CO_2_ enhances the formation, nutrient scavenging and drug resistance properties of *C. albicans* biofilms

**DOI:** 10.1038/s41522-021-00238-z

**Published:** 2021-08-12

**Authors:** Daniel R. Pentland, Jack Davis, Fritz A. Mühlschlegel, Campbell W. Gourlay

**Affiliations:** 1 Kent Fungal Group, School of Biosciences, University of Kent, Kent, UK; 2 Laboratoire National de Santé, Dudelange, Luxembourg

**Keywords:** Pathogens, Biofilms, Cellular microbiology

## Abstract

*C. albicans* is the predominant human fungal pathogen and frequently colonises medical devices, such as voice prostheses, as a biofilm. It is a dimorphic yeast that can switch between yeast and hyphal forms in response to environmental cues, a property that is essential during biofilm establishment and maturation. One such cue is the elevation of CO_2_ levels, as observed in exhaled breath for example. However, despite the clear medical relevance, the effect of CO_2_ on *C. albicans* biofilm growth has not been investigated to date. Here we show that physiologically relevant CO_2_ elevation enhances each stage of the *C. albicans* biofilm-forming process: from attachment through maturation to dispersion. The effects of CO_2_ are mediated via the Ras/cAMP/PKA signalling pathway and the central biofilm regulators Efg1, Brg1, Bcr1 and Ndt80. Biofilms grown under elevated CO_2_ conditions also exhibit increased azole resistance, increased Sef1-dependent iron scavenging and enhanced glucose uptake to support their rapid growth. These findings suggest that *C. albicans* has evolved to utilise the CO_2_ signal to promote biofilm formation within the host. We investigate the possibility of targeting CO_2_-activated processes and propose 2-deoxyglucose as a drug that may be repurposed to prevent *C. albicans* biofilm formation on medical airway management implants. We thus characterise the mechanisms by which CO_2_ promotes *C. albicans* biofilm formation and suggest new approaches for future preventative strategies.

## Introduction

*Candida albicans* is a commensal yeast located on the mucosal surfaces of the oral cavity, gastrointestinal tract and genitourinary tract of most healthy individuals^1,2^. Despite being a commensal organism, it is also an opportunistic pathogen^1,3^; in fact, it is the most widespread of all the human fungal pathogens^4^ and is the fourth most common cause of hospital-acquired infections in the USA^1^. Infection with *C. albicans* is a particular problem among immunocompromised individuals or persons with implanted medical devices such as catheters or voice prostheses (VPs)^5,6^ upon on which the yeast grows as a biofilm^7^.

Biofilms are structured communities of microorganisms attached to a surface. The cells are often encased within an extracellular matrix (ECM), which is commonly comprised of DNA^8,9^, lipids^8^, proteins^8,10^ and polysaccharides^8^. *C. albicans* is able to form biofilms on both abiotic and biotic surfaces and biofilm-associated cells are considerably more resistant to traditional antifungals when compared to planktonic cells^11^. The reasons for this increased resistance are complex but include the presence of an ECM that can act as a barrier to prevent antimicrobial agents reaching the cells^12,13^, the presence of metabolically dormant persister cells inherent to biofilms^14^ and the upregulation of drug efflux pumps^15^. A significant percentage of human microbial infections arise from or are mediated via the formation of a biofilm^16–18^, and this, combined with the limited treatment options available, means the ability of *C. albicans* to grow as a biofilm is of particular medical interest.

*C. albicans* is a dimorphic fungus, and it has the ability to undergo a morphogenic switch from a yeast to pseudohyphal or hyphal forms in response to environmental cues. The virulence of *C. albicans* is closely linked with the capacity to switch between these forms: hyphal *C. albicans* cells are frequently located at sites of tissue invasion, and cells that are unable to readily form hyphae exhibit reduced virulence^1^. The yeast-to-hyphal switch is also critical to biofilm formation as hyphal cells express a number of specific cell surface adhesins that enable cell–cell and cell–surface attachment^19^.

*C. albicans* biofilm formation is a complex process involving tightly regulated, interwoven signalling pathways centrally controlled by a set of nine transcription factors (TFs): Bcr1, Brg1, Efg1, Flo8, Gal4, Ndt80, Rob1, Rfx2, and Tec1^20,21^. These nine essential regulators function at different stages throughout *C. albicans* biofilm formation^22^ and coordinate the expression of >1000 target genes upregulated during biofilm formation^20^. Please see Lohse et al.^23^ for a comprehensive review of *C. albicans* biofilms.

Elevated CO_2_ levels, as found in a number physiologically relevant scenarios such as in exhaled breath or hypercapnia, have been shown to promote the yeast-to-hyphal switch in *C. albicans*. CO_2_ is converted to bicarbonate ions HCO_3_^−^ by the enzyme carbonic anhydrase, which in turn activate the adenylate cyclase Cyr1, resulting in increased cAMP levels and the protein kinase A (PKA)-dependent activation of hyphal specific genes^24^. The yeast-to-hyphal switch is critical to the biofilm maturation process of *C. albicans*^25^, as well as being important to its virulence^1^. The effect of CO_2_ may be particularly important within the context of biofilm development on VPs or other airway management devices as they are situated in the throat of patients where they are consistently exposed to high CO_2_ (5%) levels during exhalation. If CO_2_ does play a role in *C. albicans* biofilm maturation, it could offer a possible explanation as to why *C. albicans* is found in such high frequencies on failed VPs. In addition, CO_2_ content within the blood is also elevated (46 and 40 mmHg for venous and arterial blood, respectively, vs. 0.3 mmHg found in atmospheric air)^26,27^, and it has been estimated that as many as 80% of all microbial infections directly or indirectly involve pathogenic biofilms^28^. Thus, the work presented here could be more widely applicable to bloodstream infections and biofilm formation within the body.

Here we demonstrate that physiologically relevant increases in CO_2_ accelerate *C. albicans* biofilm formation on silicone surfaces. Transcriptome analysis reveals that several core biofilm regulatory pathways, including those governed by Efg1, Bcr1, Brg1 and Ndt80, are upregulated. We also demonstrate that a high CO_2_ environment results in increased resistance of biofilms to azole antifungals, enhanced dispersal of cells from mature biofilms and an increase in capacity for glucose uptake. Moreover, a transcription factor knockout (TFKO) library screen demonstrated TFs involved in the acquisition of iron, such as the HAP TFs Hap43, Hap2, Hap3 and Hap5, to be important for *C. albicans* biofilm formation on silicone surfaces in atmospheric CO_2_ conditions. However, high CO_2_ was able to overcome the requirement for HAP TF activity and enable *C. albicans* biofilms to forage for essential metabolites to support growth. Overall, we propose that *C. albicans* has adapted to utilise the high CO_2_ environment found in the host to promote its ability to colonise and to compete for nutrition. Our analysis reveals new approaches that can be taken to prevent *C. albicans* biofilm formation in high CO_2_ environments that pave the way for new therapeutic approaches to treat these highly drug-resistant structures.

## Results

### CO_2_ enhances *C. albicans* biofilm growth

**Fig. 1:**
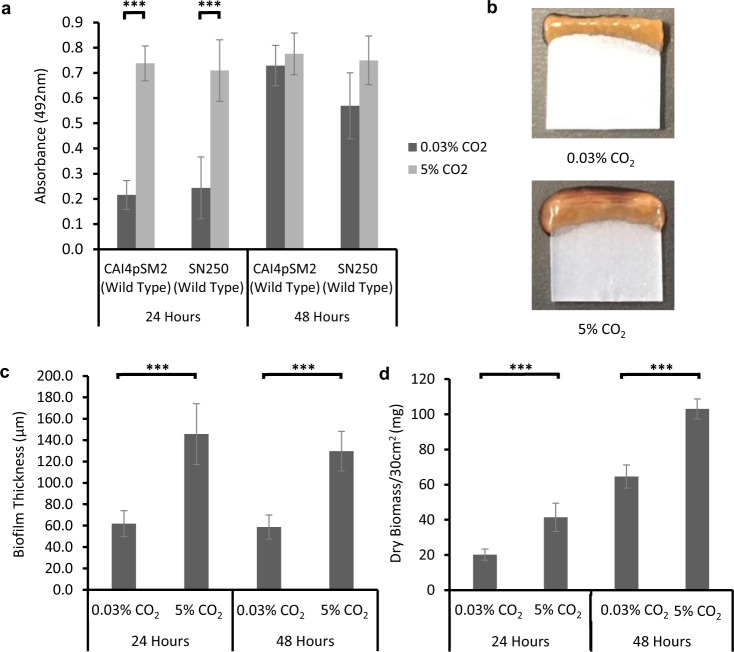
The effect of high CO_2_ (5%) on *C. albicans* biofilm formation. **a** Reference strain biofilms were seeded and grown for 24 or 48 h in 0.03% or 5% CO_2_; the resulting biofilms were quantified using the XTT assay with absorbance at 492 nm as a readout. **b** Representative images of *C. albicans* (SN250 strain) biofilms grown in 0.03% and 5% CO_2_ for 48 h (red colouration due to XTT assay). **c** SN250 biofilms were seeded on chamber slides and grown for 24 or 48 h in 0.03% or 5% CO_2_ before staining with SPYRO Ruby biofilm matrix stain. *Z*-stacks were taken using ×20 objective magnification and biofilm thickness was quantified. Graphs represent three biological replicates, error bars denote standard deviation. Paired two-tail *t* tests were carried out: ***p < 0.001, n.s. = not significant.

*C. albicans* biofilms were seeded and grown on silicone in CO_2_ levels found in exhaled air (5%) and atmospheric air (0.03%) for 24 and 48 h. Biofilms were quantified using the XTT assay, which is a metabolic dye that acts as a readout of viable cell number^29^. After 24 h of growth, both CAI4pSM2 and SN250 *C. albicans* strains exhibited a significantly higher absorbance at 492 nm for biofilms grown in a 5% CO_2_. However, after 48 h, biofilms grown in both CO_2_ conditions had equivalent 492 nm absorbance (Fig. 1a). Interestingly, although XTT readout appeared comparable at 48 h, the resultant biofilm mass was noticeably larger in biofilms grown under elevated CO_2_ conditions (Fig. 1b). We quantified this larger biofilm mass via confocal scanning laser microscopy (CSLM) and dry biomass assays. CSLM showed a significantly increased biofilm thickness when grown in 5% CO_2_ at both 24 and 48 h growth (Fig. 1c). Unlike cell number, the overall biofilm thickness did not increase between 24 and 48 h in either CO_2_ environment (Fig. 1c). However, the dry biomass of SN250 biofilms grown in 5% CO_2_ was significantly higher compared to those grown in 0.03% CO_2_ at both 24 and 48 h growth (Fig. 1d). Importantly, these increases in growth appear to be a biofilm-specific phenomenon, and not simply a result of increased growth rate in elevated CO_2_ conditions, as planktonic growth assays showed no significant difference between *C. albicans* generation time in 0.03 and 5% CO_2_ environments (Supplementary Fig. S1).

### Analysis of the effects of CO_2_ on phases of *C. albicans* biofilm growth

**Fig. 2:**
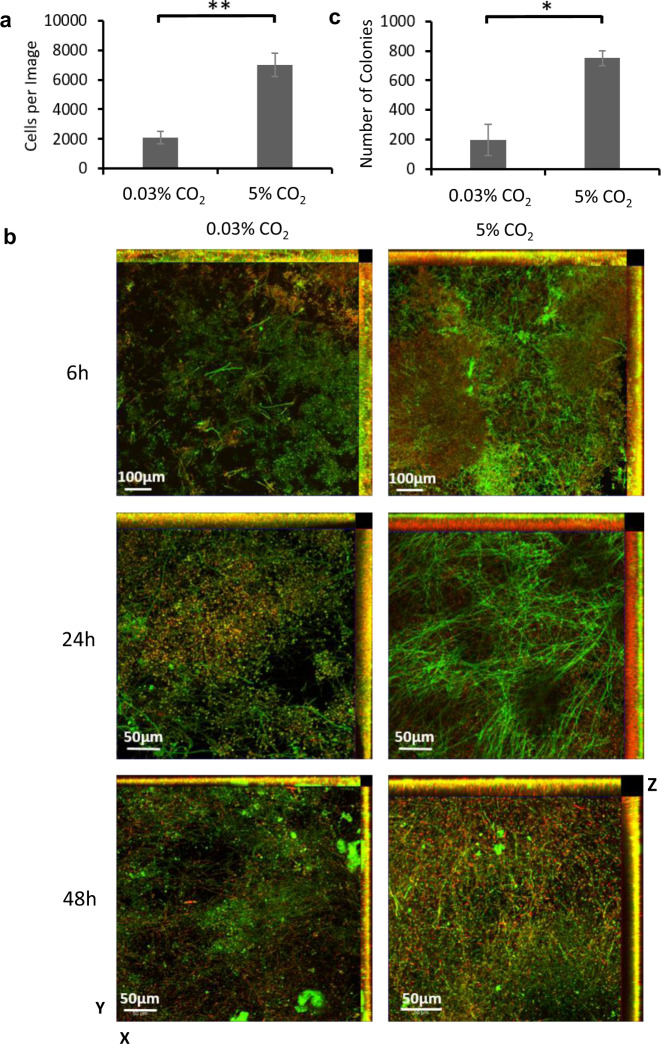
The effects of CO_2_ on *C. albicans* biofilm growth. **a** Attachment: *C. albicans* CAI-4 cells were seeded onto silicone-coated microscope slides under 0.03% or 5% CO_2_ and images were taken at ×20 objective magnification. Cells per image were counted and the mean was calculated across three biological replicates (five images per replicate). A paired two-tail *t* test was carried out: **p < 0.01. Error bars denote standard deviation. **b** Maturation: Biofilms were seeded on silicone-coated microscope slide and grown for 6, 24 and 48 h. Biofilms were stained with ConA-FITC (green) and FUN-1 (red). *Z*-stack images were taken using ×20 (6 h) and ×40 (24 and 48 h) magnifications. Experiments were repeated in triplicate and representative maximum intensity images are presented as well as *Z*-stack profiles. **c** Dispersion: Spent media was collected from biofilms grown for 48 h in 0.03% or 5% CO_2_ and diluted 1:10 before being plated to assess the number of colonies. Three biological replicates each containing technical triplicates were conducted, error bars denote standard deviation. A paired two-tail *t* test was carried out: *p < 0.05.

We sought to determine which phases of biofilm growth were influenced by CO_2_ elevation. To investigate the attachment phase *C. albicans* cells were seeded for 90 min onto silicone-coated microscopic slides under 0.03% CO_2_ or 5% CO_2_ levels. We observed that exposure to elevated CO_2_ led to a significant increase in the number of cells that attached to silicone (mean of 2108 cells in 0.03% vs. mean of 7033 cells in 5% CO_2_) (Fig. 2a). Cells also appeared to attach as larger aggregations in 5% CO_2_ when compared to those in 0.03% CO_2_ (Supplementary Figs. S2A, B) indicating that both cell–substrate and cell–cell attachments may be enhanced. These cell aggregations were not the consequence of cell wall stress. *C. albicans* exhibited equivalent sensitivities to the cell wall stressors Congo Red and Calcofluor White in both atmospheric and 5% CO_2_ environments (Supplementary Fig. S3A), with no statistically significant difference in lag time duration or generation time at each stressor concentration in a planktonic growth assay (Supplementary Fig. S3B).

CSLM was used to investigate the effects of CO_2_ on biofilm growth during maturation. *C. albicans* biofilms were seeded on silicone-coated microscopic slides and images were taken at 6, 24 and 48 h of growth under either 0.03% or 5% CO_2_ growth conditions (Fig. 2b). Biofilm images are displayed as maximum intensity ortho-projections of *Z*-stacks to give a view of the overall structures of the biofilms. After 6 h growth in 0.03% CO_2_, the majority of cells were found in the yeast form with some visibly initiating hyphae. In comparison, biofilms grown in 5% CO_2_ appeared to consist of a high proportion of hyphal cells, were visibly denser and had begun to exhibit an ordered structure in the *Z*-plane (Fig. 2b). After 24 h growth in 0.03% CO_2_, biofilms were progressing through the maturation stage with the appearance of numerous hyphal cells. However, the 5% CO_2_ biofilms exhibited a fully mature biofilm organisation displaying hyphal cells organised in a brush-like structure above a basal layer of yeast cells (Fig. 2b). At 48 h, biofilms grown in both 0.03% and 5% CO_2_ appeared as dense mature structures; however, biofilms grown under elevated CO_2_ appeared larger (Fig. 2b) as had been observed previously (Fig. 1b, c).

Dispersion is the final stage of biofilm formation, we therefore investigated whether CO_2_ elevation resulted in increased levels of cell shedding. We routinely observed that spent RPMI-1640 media isolated after biofilm growth in 5% CO_2_ contained more cells than that of taken from biofilms grown in 0.03% CO_2_ (Supplementary Fig. S3A). We quantified this by seeding and growing *C. albicans* biofilms for 48 h in 0.03% and 5% CO_2_ and conducting colony-forming unit assays using the spent RPMI-1640 media (Fig. 2c and Supplementary Fig. S4B). Our results showed an approximate fourfold increase in cell number released from mature biofilms when grown under elevated CO_2_ conditions, consistent with an increase in dispersal. Overall, these data demonstrate that the elevation of CO_2_ enhances each stage of the *C. albicans* biofilm-forming process, from attachment through maturation to dispersion.

### Identification of the regulatory mechanisms that govern CO_2_ acceleration of *C. albicans* biofilm formation

**Fig. 3:**
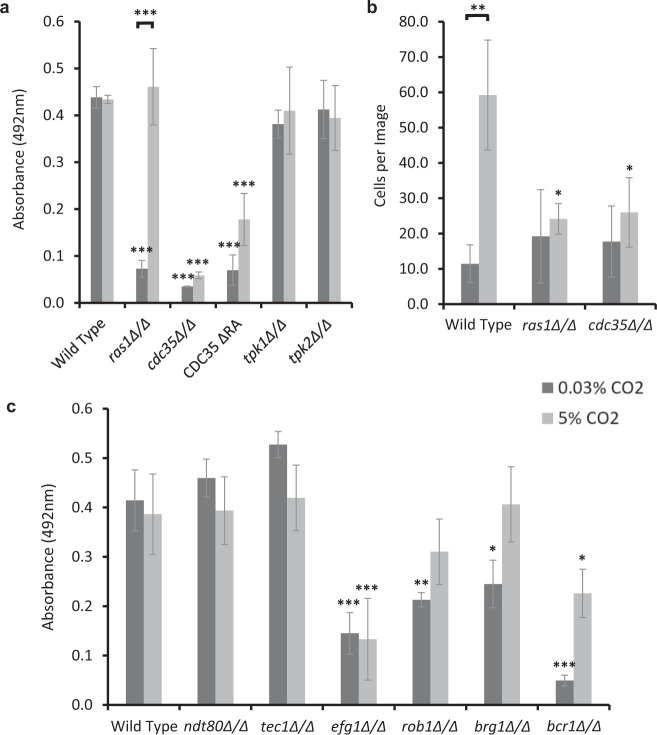
Biofilm growth assays of *C. albicans* Ras1-Cyr1-PKA pathway and central biofilm regulator null mutants. Cells were seeded and counted or grown as biofilms for 48 h before XTT quantification. **a** Biofilm growth assay using Ras1-Cyr1(Cdc35)-PKA pathway null mutants. **b** Attachment assay on silicone surface using *ras1Δ/Δ* and *cdc35Δ/Δ* mutants. **c** Biofilm growth assay using central biofilm regulator null mutants. Graphs represent three biological replicates, error bars denote standard deviation. Two-way ANOVAs followed by Tukey tests for multiple comparisons were carried out: *p < 0.05, **p < 0.01, ***p < 0.001. Asterisks directly above the bars indicate a significant difference to the wild type in the same CO_2_ environment.

In planktonic *C. albicans* cells, CO_2_ is converted to bicarbonate ions (HCO_3_^−^), which stimulates the adenylate cyclase Cyr1 (Cdc35), causing an increase in cAMP and activation of PKA^24^. We investigated whether CO_2_ elevation may drive biofilm formation and maturation via a similar Ras/cAMP/PKA mechanism. We conducted biofilm growth assays using *C. albicans* mutants lacking key components of the pathway: *ras1Δ/Δ*, *cdc35Δ/Δ*, *CDC35*^ΔRA^ (adenylate cyclase missing the Ras1 interacting domain), *tpk1Δ/Δ* (missing a catalytic subunit isoform of PKA), and *tpk2Δ/Δ* (missing the other catalytic subunit isoform of PKA). Biofilm formation was quantified after 48 h of growth and compared to an isogenic wild-type control. The *ras1Δ/Δ* mutant displayed significantly attenuated biofilm growth in 0.03% CO_2_ but this was rescued to wild-type levels in 5% CO_2_ (Fig. 3a), indicating that Ras1 function is dispensable for biofilm formation under conditions of elevated CO_2_. The *cdc35Δ/Δ* and *CDC35*^ΔRA^ mutants both exhibited significantly reduced biofilm growth when grown under either atmospheric or elevated CO_2_ conditions (Fig. 3a). Both *tpk1Δ/Δ* and *tpk2Δ/Δ* mutants exhibited biofilm growth equivalent to the wild type under both CO_2_ conditions (Fig. 3a). To further explore the *ras1Δ/Δ* and *cdc35Δ/Δ* biofilm phenotypes, we conducted silicone surface attachment assays. Contrary to the biofilm growth assays, attachment of both mutants was similar to the wild type in 0.03% CO_2_ (Fig. 3b). Significantly fewer *cdc35Δ/Δ* cells than wild type attached to silicone in 5% CO_2_, interestingly, despite its wild-type levels of biofilm formation in 5% CO_2_; this was also the case for the *ras1Δ/Δ* mutant (Fig. 3b). Taken together, this data implies that both Ras1 and Cyr1 are required for CO_2_-mediated attachment increase, while the CO_2_ effect on *C. albicans* biofilm growth is reliant on Cyr1 but can bypass a requirement for Ras1. Moreover, under these experimental conditions, the PKA isoforms Tpk1 and Tpk2 are functionally redundant with respect to cAMP activation of the biofilm programme; however, we are not able to discount the possibility that Cyr1 may direct biofilm formation independently of PKA activation. Our findings are consistent with the adenylate cyclase Cyr1 as a key CO_2_ sensor in the enhanced biofilm growth observed under elevated CO_2_ conditions.

To investigate the molecular basis of the activation of *C. albicans* biofilm formation in 5% CO_2_, we conducted a screen of an available TFKO library^30^ containing 147 mutants each lacking a non-essential TF. This screen identified 122 deletions that had no effect upon biofilm formation in either CO_2_ condition (Supplementary Fig. S5) and 22 TFs, which attenuated biofilm formation (Supplementary Table S2). Six TFKO mutants (*tup1Δ/Δ*, *sef1Δ/Δ*, *swi4Δ/Δ*, *pho4Δ/Δ, bcr1Δ/Δ* and *efg1Δ/Δ*) had diminished biofilm growth in both 0.03% and 5% CO_2_, 12 had reduced biofilm formation only in 0.03% CO_2_ (*hap2Δ/Δ*, *rbf1Δ/Δ*, *rob1Δ/Δ*, *fgr15Δ/Δ*, *dal81Δ/Δ*, *mig1Δ/Δ*, *brg1Δ/Δ*, *C4_00260WΔ/Δ*, *zcf27Δ/Δ*, *C1_13880CΔ/Δ*, *crz1Δ/Δ* and *hap43Δ/Δ*), and 4 TFKOs had reduced biofilm formation only in 5% CO_2_ (*leu3Δ/Δ*, *mbp1Δ/Δ*, *bas1Δ/Δ* and *try6Δ/Δ*) (Supplementary Table S2 and Supplementary Fig. S5). Intriguingly, 3 additional mutants, *zcf17Δ/Δ*, *zcf30Δ/Δ* and *mac1Δ/Δ*, displayed increased biofilm growth in 0.03% CO_2_ but no significant difference in 5% CO_2_.

We performed gene ontology (GO) enrichment analysis on the genes encoding the 25 TFs whose loss impacted upon biofilm growth to group them according to biological processes. This revealed that seven of these TFs—Brg1, Bcr1, Efg1, Rob1, Dal81, Leu3 and Try6—were already known to be involved in the regulation of single-species biofilm formation within *C. albicans* (Supplementary Table S2). Interestingly, out of these, only the *efg1Δ/Δ* and *bcr1Δ/Δ* mutant exhibited attenuated biofilm growth in both 0.03% and 5% CO_2_. The *brg1Δ/Δ*, *dal81Δ/Δ* and *rob1Δ/Δ* mutants had significantly reduced biofilm growth in 0.03% CO_2_, but this was rescued in the 5% CO_2_ environment (Supplementary Table S2), possibly indicating redundancy of these TFs when cells are exposed to high CO_2_. This important finding suggests that CO_2_ elevation can bypass the requirement for some of the key transcriptional regulators of biofilm formation (Fig. 3c). The *leu3Δ/Δ* and *try6Δ/Δ* mutants had significantly reduced biofilm growth in 5% CO_2_ but no significant difference in 0.03% CO_2_. Eight of the TFs identified—Brg1, Crz1, Efg1, Mac1, Mig1, Rob1, Zcf27, Zcf30—are associated with the positive regulation of filamentous growth and Tup1 is involved in the negative regulation of filamentous growth. *C. albicans* biofilm formation is strongly linked with the yeast-to-hyphal switch, with mutants unable to perform this switch having previously been shown to be deficient in biofilm growth ^25^.

### Regulation of iron homoeostasis in *C. albicans* biofilms

**Fig. 4:**
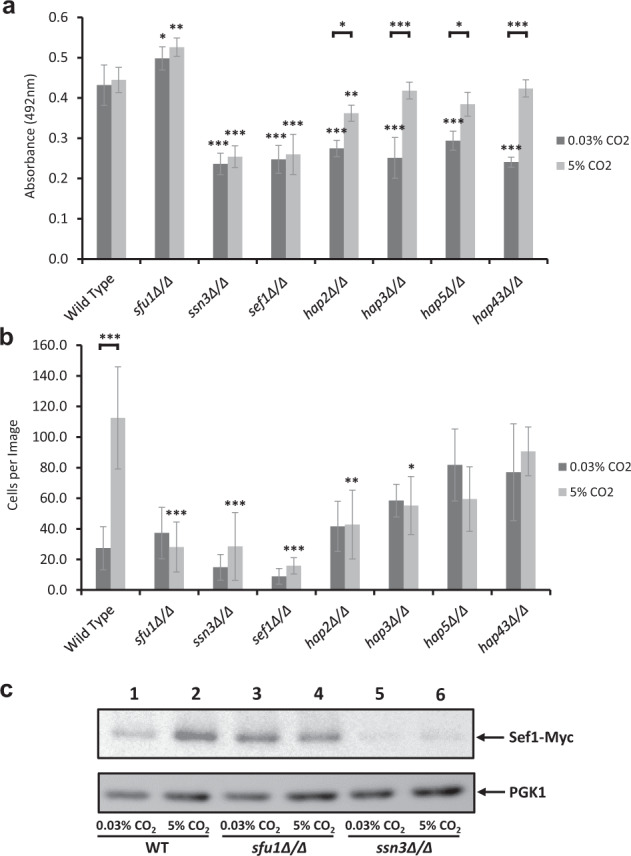
The effect of high (5%) CO_2_ on iron homoeostasis in *C. albicans* biofilms. **a** Biofilm growth assay using iron homoeostatic pathway null mutants. Biofilms were seeded and grown for 48 h before XTT quantification. **b** Iron homoeostatic mutants were seeded on a silicone surface and the number of attached cells were quantified from microscopic images. Graphs represent at least three biological replicates each containing technical triplicates, error bars denote standard deviation. Two-way ANOVAs followed by Tukey tests for multiple comparisons were carried out: *p < 0.05, **p < 0.01, ***p < 0.001. **c** Immunoblot of Sef-Myc and PGK1 (internal standard) from 48 h biofilms in wild-type, *sfu1Δ/Δ*, and *ssn3Δ/Δ* backgrounds.

The TFKO screen also revealed that mutants lacking TFs associated with metal ion homoeostasis, specifically iron homoeostasis, had altered biofilm formation in 0.03% and/or 5% CO_2_ (Fig. 4a and Supplementary Table S2). Mutants lacking genes expressing components of the HAP complex: *hap2Δ/Δ*, *hap3Δ/Δ*, *hap5Δ/Δ* and *hap43Δ/Δ*, exhibited significantly reduced biofilm growth after 48 h compared to wild type in 0.03% CO_2_. However, their biofilm growth was significantly higher in 5% CO_2_, reaching wild-type levels in the cases of the *hap3Δ/Δ*, *hap5Δ/Δ* and *hap43Δ/Δ* mutants (Fig. 4a). This is an important observation as it indicates that an increase in CO_2_ concentration is sufficient to compensate for the loss of these TFs. The HAP complex in *C. albicans* is responsible for the regulation of iron homoeostasis^31^. Sef1 acts downstream of this complex as an activator of iron uptake genes and the *sef1Δ/Δ* mutant displayed significantly reduced biofilm growth in both 0.03% and 5% CO_2_ after 48 h growth (Fig. 4a). The transcriptional repressor Sfu1 and the kinase Ssn3 are negative and positive regulators of Sef1 activity, respectively^32^; the *sfu1Δ/Δ* mutant showed significantly increased biofilm growth compared to wild type in both CO_2_ environments, while the *ssn3Δ/Δ* mutant presented an identical phenotype to *sef1Δ/Δ* (Fig. 4a). These data suggest that the elevation of CO_2_ can bypass the requirement for HAP complex activity in biofilm formation in a Sef1-dependent manner.

Intriguingly, in attachment assays on a silicone surface, none of these iron homoeostatic mutants showed a statistically significant difference in attachment compared to wild type in 0.03% CO_2_ (Fig. 4b). However, the *sfu1Δ/Δ*, *ssn3Δ/Δ*, *sef1Δ/Δ*, *hap2Δ/Δ* and *hap3Δ/Δ* mutants all attached in significantly lower numbers than wild type in 5% CO_2_, suggesting a role for iron homoeostatic control in the CO_2_-dependent attachment increase. Furthermore, unlike mature biofilm quantification, none of the HAP complex mutants displayed a CO_2_-dependent attachment phenotype (Fig. 4b).

Immunoblot analysis of Myc-tagged Sef1 recovered from 48 h biofilms in wild-type, *sfu1Δ/Δ* and *ssn3Δ/Δ* backgrounds revealed that Sef1 levels were higher in wild-type mature biofilms grown in 5% CO_2_ than those grown in atmospheric CO_2_ (Fig. 4c, lanes 1 and 2). Sef1 was present in mature biofilms regardless of environmental CO_2_ levels in the absence of Sfu1 (Fig. 4c, lanes 3 and 4). However, in the absence of Ssn3, Sef1 expression was low in either CO_2_ condition (Fig. 4c). These data are not only consistent with Sfu1 and Ssn3 being negative and positive regulators, respectively, of Sef1, as demonstrated in planktonic cells by Chen et al.^32^, but also suggest a role for a CO_2_ in activating iron uptake pathways in *C. albicans* biofilms in an Ssn3-dependent manner.

**Fig. 5:**
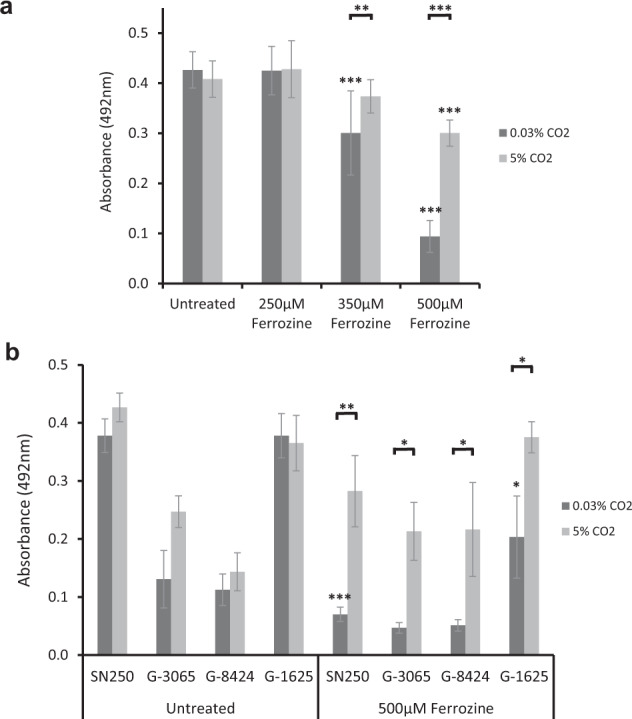
The effect of high (5%) CO_2_ on iron starvation in *C. albicans* biofilms. Biofilms were seeded and grown for 48 h in the presence of the Fe^2+^ chelator Ferrozine before XTT quantification. **a** Iron starvation biofilm growth assay using the SN250 reference strain. Graph represents six biological replicates each containing technical triplicates, error bars denote standard deviation. **b** Iron starvation biofilm growth assay using clinical isolates. Graph represents three biological replicates each containing technical triplicates, error bars denote standard deviation. Two-way ANOVAs followed by Tukey tests for multiple comparisons were carried out: *p < 0.05, **p < 0.01, ***p < 0.001.

To further determine whether elevated CO_2_ enhanced *C. albicans* ability to appropriate iron from the environment, biofilms were grown in the presence of the Fe^2+^ chelator Ferrozine. Iron chelation was observed to have a marked effect on biofilm growth at and above 350 μM Ferrozine in RPMI media (Fig. 5a). As had been observed with TFKO strains of the HAP complex, the elevation of CO_2_ counteracted the effects of iron limitation (Fig. 5a), providing further evidence of a role for CO_2_ in enhancing iron uptake or utilisation capability. This tolerance to iron starvation of *C. albicans* biofilms grown in 5% CO_2_ was also exhibited by *tpk1Δ/Δ* and *tpk2Δ/Δ* mutants (Supplementary Fig. S5), indicating that these PKA isoforms are functionally redundant with respect to iron homoeostatic pathways. Clinical strains of *C. albicans* isolated from failed VPs displayed the same phenotype with biofilm formation rescued in 5% CO_2_ when grown in the presence of 500 μM Ferrozine (Fig. 5b). A transcriptomic analysis comparing biofilms grown in 0.03% and 5% CO_2_ (described below) revealed that several genes related to iron acquisition were upregulated in 5% CO_2_ biofilms, providing an explanation for the tolerance to iron sequestration in high CO_2_.

### Transcriptome analysis of *C. albicans* biofilms grown in high and low CO_2_

**Fig. 6:**
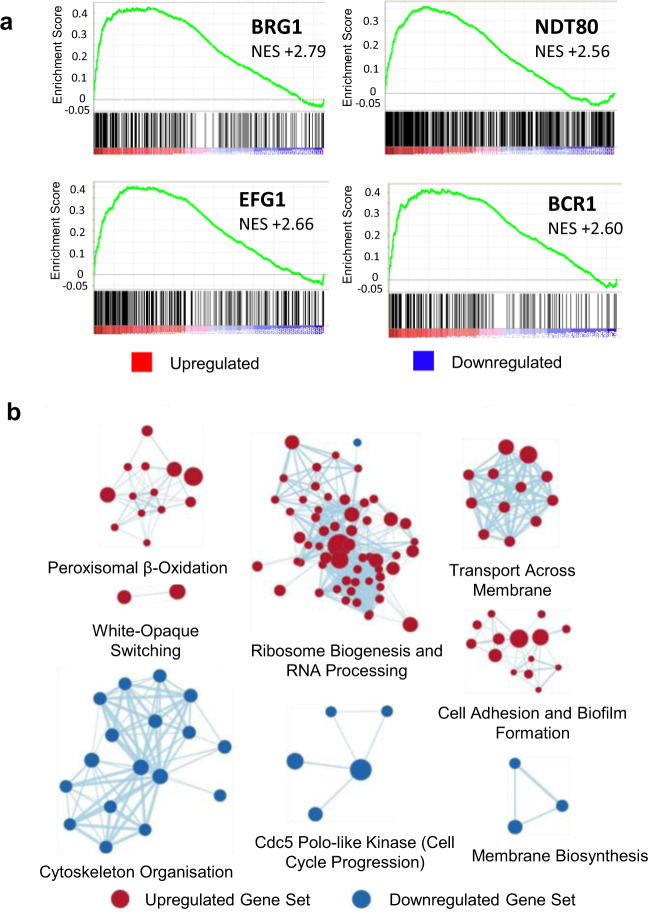
Global gene expression changes in 5% CO_2_ vs. 0.03% CO_2_*C*. *albicans* biofilms. **a** GSEA enrichment plots of central biofilm regulator gene sets with altered expression levels as assessed by RNA Sequencing; four of the nine identified core regulators of biofilm formation (Brg1, Efg1, Ndt80, and Bcr1)^20^ were identified as having positive GSEA scores. Vertical black lines represent individual genes in the significantly differentially expressed ranked gene list from upregulated (left) to downregulated (right). The enrichment score increases if there are lots of genes towards the beginning of the ranked list (upregulated). NES normalised enrichment score; positive NES indicates enrichment in the upregulated group of genes. **b** Gene set cluster map showing the most upregulated and downregulated gene sets as determined by GSEA along with their cellular functions. Each circle is a gene set and the lines between them depict how much they overlap, thicker line = more genes in common.

To further investigate the effect of high CO_2_ on *C. albicans* biofilm growth, we performed an RNA Sequencing (RNA-Seq) analysis of *C. albicans* biofilms grown in 0.03% and 5% CO_2_. Growth in 5% CO_2_ led to a global response resulting in the significant differential expression (false discovery rate (FDR) adjusted *p* value (*q*) ≤0.05) of 2875 genes, with roughly equal numbers of genes upregulated and downregulated (1441 up and 1434 down) (Supplementary Fig. S7A). Eighty genes were strongly (log2 fold change >2) upregulated and 25 were strongly (log2 fold change <−2) downregulated. To investigate the cellular pathways which these differentially expressed genes are within, we conducted Gene Set Enrichment Analysis (GSEA; Broad Institute)^33^. GSEA revealed that biofilm formation pathways controlled by four of the nine ‘core’ biofilm regulator TFs^20,21^ were upregulated in 5% CO_2_ biofilms at 48 h (Fig. 6a). Genes downstream of Brg1, Efg1, Ndt80 and Bcr1 are enriched in the upregulated genes at the top of the ranked list of differentially expressed genes (normalised enrichment score (NES) = +2.79, +2.66, +2.56 and +2.60, respectively; Fig. 6a), indicating that high CO_2_ drives the expression of genes previously described as important in the biofilm-forming ability of *C. albicans*.

As GSEA can show enrichment profiles exhibiting correlations with several overlapping gene sets, we visualised networks of similar gene sets using the Cytoscape 3.7.1 EnrichmentMap plug-in^34^. Upregulated gene sets included those encoding membrane transporters, ribosome biogenesis, peroxisomal β-oxidation and white-opaque switching (Fig. 6b). Pathways involved in cytoskeleton organisation, cell-cycle progression and membrane biosynthesis were downregulated in high CO_2_ conditions (Fig. 6b), indicating that cells within biofilms in high CO_2_ may have stopped dividing at the 48 h time point assessed, consistent with such biofilms having moved more rapidly through the maturation process.

**Fig. 7:**
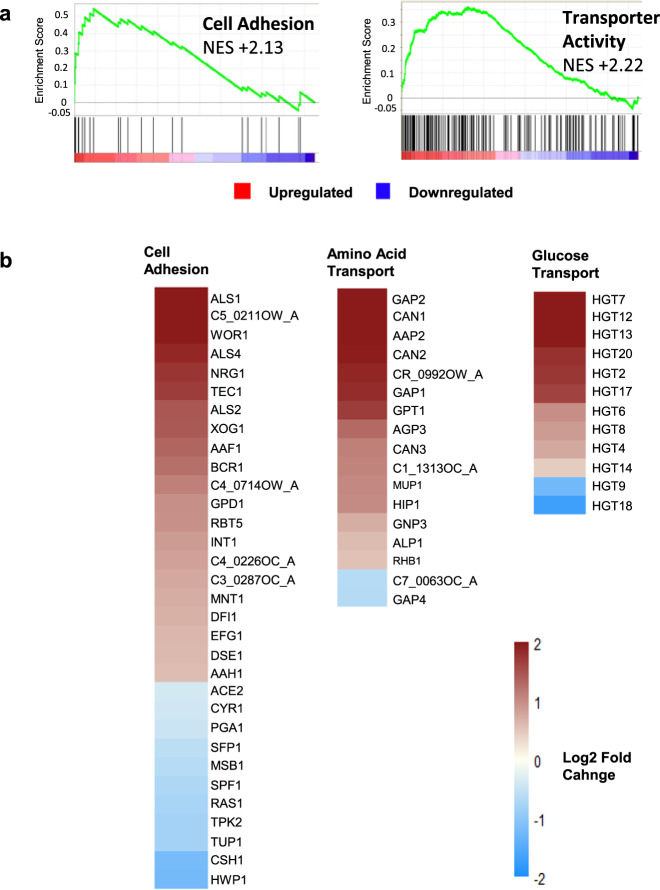
Adhesion and transport processes are upregulated in 5% CO_2_*C*. *albicans* biofilms. **a** GSEA enrichment plot of the BIOLOGICAL ADHESION_BIO and TRANSPORTER ACTIVITY_MOL, AMINO ACID TRANSPORT_BIO and CARBOHYDRATE TRANSPORTER ACTIVITY_MOL gene sets containing genes under the GO terms ‘transporter activity’ and ‘amino acid transport’. NES normalised enrichment score; positive NES indicates enrichment in the upregulated group of genes. **b** Heat map of significantly differentially expressed genes associated with cell adhesion, amino acid transport and glucose transport as identified by GO Slim process analysis. The colours saturate at log2 fold change of 2 and −2.

GSEA suggested that cell adhesion processes were upregulated in high CO_2_ biofilms (NES = +2.13; Fig. 7a). GO Slim process analysis also highlighted that many genes associated with cell adhesion were upregulated in *C. albicans* biofilms grown in 5% CO_2_ after 48 h (Fig. 7b). The second highest upregulated gene in 5% CO_2_ biofilms compared to 0.03% CO_2_ biofilms was *ALS1* with a 3.77 log2 fold change (Fig. 7b). The Als1 cell surface adhesin has previously been shown to have important roles in biofilm formation^35^, and its expression is controlled by the biofilm transcription regulation network composed of Brg1, Rob1, Tec1, Ndt80, Bcr1 and Efg1^20^. Other genes encoding cell surface adhesins such as *ALS4* and *ALS2* were also upregulated in 5% CO_2_ biofilms after 48 h (Fig. 7b).

GSEA has also identified that genes involved in membrane transporter activity were enriched in the upregulated genes at the top of the ranked list of differentially expressed genes (NES = +2.22; Fig. 7a). Specifically, genes encoding amino acid transporters were enriched in the significantly upregulated genes of 5% CO_2_ biofilms (NES = +2.62). Likewise, significantly differentially expressed genes possessing the GO term ‘amino acid transport’ were primarily upregulated (Fig. 7b). The most highly upregulated of these was *GAP2*, which encodes a general amino acid permease^36^. *GAP2* was in fact the highest upregulated gene of the entire RNA-Seq data set with a 4.60 log2 fold change. The basic amino acid permease genes *CAN1*, *CAN2* and *CAN3* were also upregulated (Fig. 7b).

Genes associated with carbohydrate transmembrane transport were also enriched in the significantly upregulated genes of 5% CO_2_ biofilms as revealed by GSEA (NES = +1.88). Among these were 12 genes encoding putative major facilitator superfamily glucose transmembrane transporters present in the significantly differentially expressed gene list and these were almost all upregulated. The only exceptions were *HGT9* and *HGT18* with log2 fold changes of −1.24 and −1.70, respectively (Fig. 7b).

Genes previously identified to be involved in hyphal formation in response to foetal bovine serum exposure or 37 °C were enriched in the downregulated genes (NES = −3.41; Supplementary Fig. S7C). Likewise, the majority of genes under the GO term ‘hyphal growth’ in the significantly differentially expressed gene list were downregulated in 5% CO_2_ biofilms at 48 h growth (Supplementary Fig. S7B). Significantly differentially expressed genes associated with the cytoskeleton, as identified by GO term analysis, were enriched in the downregulated genes (NES = −2.69; Supplementary Fig. S7C). Cytoskeleton reorganisation is important for the growth of *C. albicans* hyphal cells^37^ as well as cell division^38,39^, indicating that cell growth is lower in 48 h-old *C. albicans* biofilms grown in 5% CO_2_ than in those grown in 0.03% CO_2_. Consistent with this, genes involved in the transition through the G1/S checkpoint were also enriched in the significantly downregulated genes (NES = −2.83; Supplementary Fig. S7C).

Initially, these data appear to be contradictory to the previous data highlighting the increased biofilm formation of *C. albicans* under high CO_2_ conditions. However, due to the increased biofilm formation in a 5% CO_2_ environment, *C. albicans* biofilms reach full maturity much quicker in high CO_2_, as observed via confocal microscopy (Fig. 2b). Thus, by 48 h *C. albicans* biofilms grown in high CO_2_ have been fully mature for several hours and hence would contain fewer dividing cells or cells extending hyphae in comparison to low CO_2_ biofilms.

### CO_2_ elevation enhances azole resistance in *C. albicans* biofilms

**Fig. 8:**
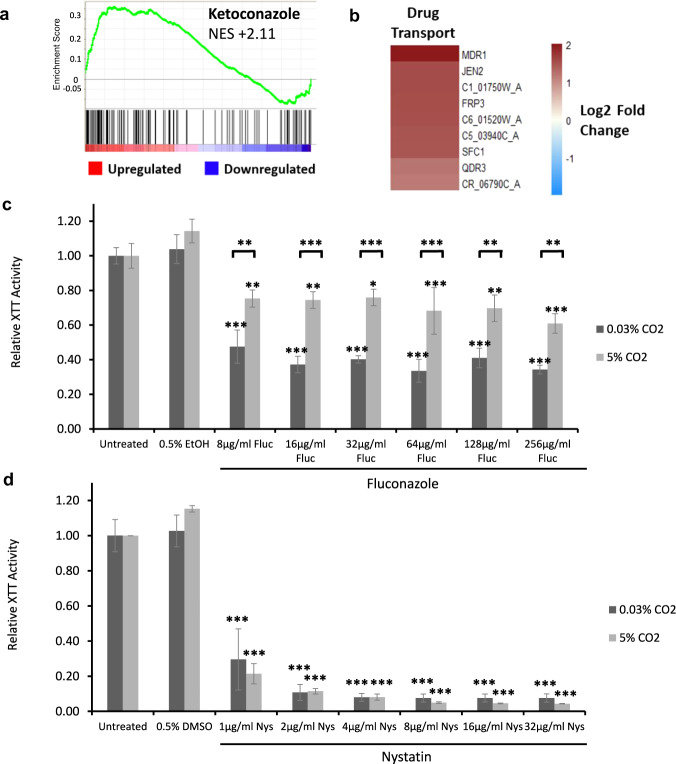
Antifungal sensitivity of *C*. *albicans* biofilms grown in high (5%) CO_2_. **a** GSEA enrichment plot of the KETOCONAZOLE_UP gene set containing genes upregulated in *C. albicans* cells grown in the presence of Ketoconazole^62^. NES normalised enrichment score; positive NES indicates enrichment in the upregulated group of genes. **b** Heat map of genes associated with drug transport, including the multidrug efflux pump gene *MDR1*. **c** Biofilm growth assay of CAI4pSM2 in the presence of Fluconazole. **d** Biofilm growth assay of CAI4pSM2 in the presence of Nystatin. The relative XTT activity is presented with the 0.03% CO_2_ biofilms being normalised to the 0.03% CO_2_ untreated control and the 5% CO_2_ biofilms being normalised to the 5% CO_2_ untreated control. Two-way ANOVAs followed by Tukey tests for multiple comparisons were carried out: *p < 0.05, **p < 0.01, ***p < 0.001. Asterisks directly above the bars indicate a significant difference to untreated in the same CO_2_ environment.

In addition to the observed increase in the expression of genes that drive biofilm formation, we observed an elevation in certain stress response pathways in *C. albicans* biofilms grown in 5% CO_2_. Gene sets involved in the response of *C. albicans* to antifungals such as Ketoconazole^40^ were predicted to be upregulated (Fig. 8a; NES = +2.11) as well as several drug transporters (Fig. 8b), indicating that elevation may lead to increased drug resistance. Upregulated genes included the *MDR1* gene (2.56 log2 fold change), which encodes the multidrug-resistance pump Mdr1 and is associated with resistance to several antifungals, such as azoles^41^. To test the significance of this, *C. albicans* biofilms were seeded and grown for 24 h in 0.03 and 5% CO_2_ before the addition of antifungals, after which they were grown for an additional 24 h in both conditions to observe the effect of drug application. Antifungal concentrations were selected based upon previously reported MIC values for these antifungals against *C. albicans* biofilms^42^. Overall, Fluconazole and Miconazole treatment led to a significant reduction in biofilm growth in 0.03% CO_2_ (Fig. 8c and Supplementary Fig. S8). Treatment with Fluconazole and Miconazole also significantly reduced biofilm formation in 5% CO_2_; however, their effects were markedly reduced (Fig. 8c and Supplementary Fig. S8). This suggests an increased resistance of biofilms grown in 5% CO_2_ to azole treatment. Interestingly, Nystatin and Caspofungin were equally as effective against biofilms in either CO_2_ environment (Fig. 8d and Supplementary Fig. S9).

### Precision approaches to overcome CO_2_ acceleration of *C. albicans* biofilm formation

**Fig. 9:**
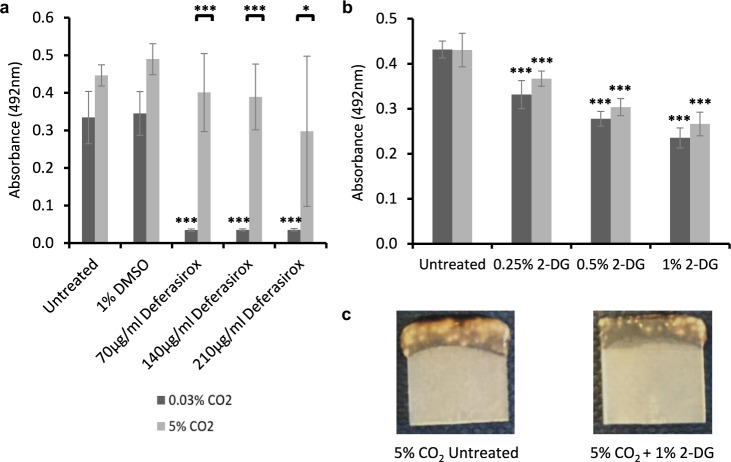
Efficacy of potential treatments to combat *C*. *albicans* biofilms grown in high (5%) CO_2_. Biofilms were seeded and grown for 48 h before XTT quantification. **a** Biofilm growth assay of SN250 in the presence of the Fe^3+^ chelator Deferasirox. Graph represents two biological replicates each containing technical triplicates, error bars denote standard deviation. **b** Biofilm growth assay of SN250 in the presence of the glycolytic inhibitor 2-DG. Graph represents three biological replicates each containing technical triplicates, error bars denote standard deviation. Two-way ANOVAs followed by Tukey tests for multiple comparisons were carried out: *p < 0.05, ***p < 0.001. Asterisks directly above the bars indicate a significant difference to the untreated SN250 in the same CO_2_ environment. **c** Representative images of SN250 biofilms grown in 5% CO_2_ ± 2-DG for 48 h.

Our data indicate that elevation of CO_2_ leads to an increase in the ability to scavenge for iron and glucose, both essential for biofilm formation and growth. We therefore wished to test whether these represented potential targets to combat *C. albicans* growth in high CO_2_ environments, such as the airway. An Fe^3+^ chelator called Deferasirox, which is approved for treating patients with iron overload, has recently been shown to reduce infection levels in a murine oropharyngeal candidiasis model^43^. With this in mind, we repeated our previous iron starvation biofilm growth assay (Fig. 5a) using Deferasirox in place of Ferrozine. We observed that Deferasirox treatment completely eradicates *C. albicans* biofilm growth in 0.03% CO_2_ but has very little effect on biofilm growth in 5% CO_2_ (Fig. 9a), thus adding further evidence that exposure to high levels of CO_2_ can enable *C. albicans* biofilms to overcome the effects of iron starvation. Deferasirox does not therefore appear to be an effective treatment against *C. albicans* biofilms in high CO_2_ such as in the context of VP colonisation.

*C. albicans* biofilms grown in 5% CO_2_ exhibited the upregulation of genes encoding glucose transporters. Accordingly, we contemplated whether treatment of *C. albicans* biofilms with the glucose analogue 2-deoxyglucose (2-DG; a glycolytic inhibitor) may decrease biofilm growth in high CO_2_ environments. 2-DG has previously made it to stage II clinical trials as an anti-prostate cancer treatment and is considered safe for use in humans^44^. Thus, it could be a potential therapeutic option to combat *C. albicans* biofilm formation on medical devices, specifically on VPs. *C. albicans* biofilm formation was significantly reduced in the presence of 2-DG regardless of CO_2_ environment (Fig. 9b and Supplementary Fig. S9). Interestingly, the biofilm reductions in all 2-DG concentrations were similar for biofilms in both low and high CO_2_, despite the fact that several glucose transporters were upregulated in 5% CO_2_ biofilms (Fig. 7b). The reduction in biofilm growth upon 2-DG treatment was also quite apparent macroscopically (Fig. 9c).

## Discussion

**Fig. 10:**
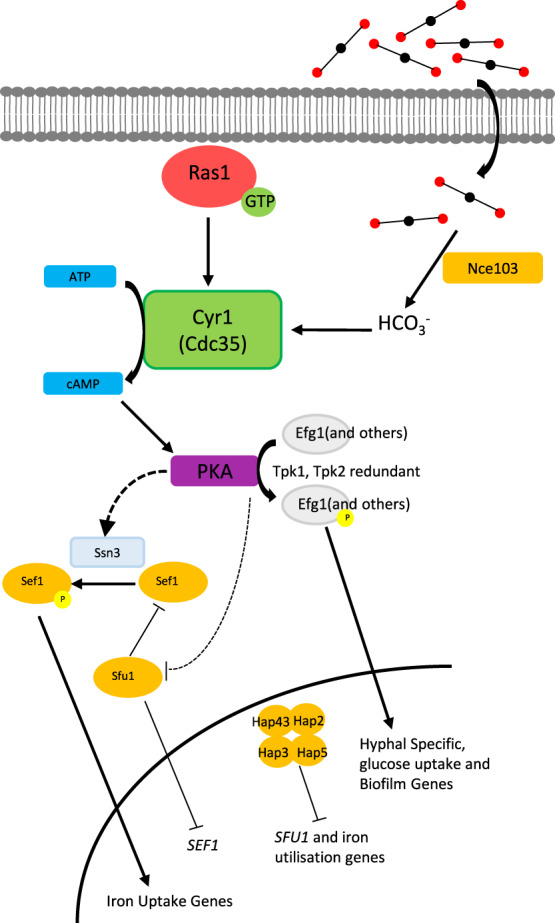
Predicted model of the interplay between CO_2_ signalling and iron homoeostasis in *C*. *albicans* biofilms on silicone surfaces. High environmental levels of CO_2_ drive biofilm formation via the Ras1-Cyr1-PKA pathway in the same way as previously shown for hyphal morphogenesis in planktonic cells. This is accompanied by an increase in iron-scavenging capability by biofilm-associated cells. Our data are consistent with a CO_2_-mediated activation of PKA via Cyr1 resulting in increased abundance and activity of Sef1 in an Ssn3-dependent manner (this process may also involve the inhibition of Sfu1). Subsequent increased expression of iron uptake genes thus enables more effective scavenging of environmental iron.

Our data demonstrate for the first time that a physiologically relevant elevation of CO_2_ accelerates biofilm formation in *C. albicans* by activating the cAMP/PKA pathway (Fig. 10). Interestingly, although CO_2_ elevation is dependent on Cyr1 it appears to bypass a requirement for Ras1. CO_2_ elevation enhances each stage of the *C. albicans* biofilm-forming process, from attachment through maturation to dispersion. The observed increase in cell attachment is accompanied by an increase in the abundance of mRNA transcripts for *ALS1*, *ALS2* and *ALS4*, which encode adhesins of the agglutinin-like sequence family that function in the cell–surface and cell–cell attachment of *C. albicans*^19^. Our observed increase in dispersion also correlates with an upregulation of the known regulator of this stage of biofilm growth, *NRG1*^45^. However, it cannot be ruled out that this is a consequence of increased biomass under elevated CO_2_ and further investigation to look for lateral yeast budding from hyphae should be carried out to confirm dispersion. Our observations have important clinical implication in scenarios where prosthetic devices are placed in areas of elevated CO_2_, for example, VPs or tracheostomy tubing. We would anticipate that high CO_2_ may increase the probability of *C. albicans* colonisation and dissemination.

Of the original six ‘core’ biofilm regulators (Efg1, Bcr1, Brg1, Rob1, Ndt80 and Tec1), only four (Efg1, Bcr1, Brg1 and Rob1) were identified as required for normal biofilm growth in our TFKO screen. The original TFKO screen that identified these regulators was carried out using a polystyrene surface^20^, whereas we used silicone. This may suggest that attachment to and biofilm growth on silicone requires a more limited set of core TFs reflecting the importance of surface upon biofilm formation as has been observed previously^21^. In support of the environment being critical to biofilm establishment, CO_2_ elevation was able to compensate for the loss of the core biofilm regulators Brg1 and Rob1. Intriguingly, of the four biofilm regulators whose gene sets were predicted to be upregulated in 5% CO_2_ biofilms, Efg1, Bcr1 and Brg1 were identified in our TFKO screen, whereas Ndt80 was not. Moreover, only *efg1Δ/Δ* and *bcr1Δ/Δ* had reduced biofilm growth in both 0.03 and 5% CO_2_. This disparity may be explained by the degree of overlap in downstream target genes between the core regulators of biofilm formation^20^, which implies significant potential for functional redundancy.

Many of the genes significantly upregulated in biofilms grown in high CO_2_ after 48 h were also found to be upregulated in other biofilm gene expression studies^46,47^. For instance, Nett et al. observed an increase in the expression of adherence genes in an in vivo venous catheter biofilm model^46^. This was also the case in a temporal gene expression analysis using in vitro denture and catheter models^47^. Interestingly, some pathways such as hexose transport, amino acid uptake and stress responses that our differential gene expression analysis predicted to be upregulated in mature biofilms grown in high CO_2_ were concluded to be upregulated only in early-phase biofilms (12 h) in a denture and catheter study^47^. The authors concluded that the result of the induction of these pathways is the increase of intracellular pools of pyruvate, pentoses and amino acids, preparing for the large increase in biomass that occurs later in biofilm development^47^. We hypothesise that high CO_2_ may stimulate these pathways and maintain their activity even in mature biofilms, thus supporting the increased biomass and maturation rate of biofilms observed when grown in high CO_2_.

We observed an increase in azole antifungal resistance within *C. albicans* biofilms grown in 5% CO_2_. This, at least partly, could be explained by the increased expression of drug transporter genes such as *MDR1*, which have previously been implicated in azole resistance^41,48^. However, *mdr1Δ/Δ*, as well as *cdr1Δ/Δ* and *cdr2Δ/Δ*, mutants only exhibit reduced azole resistance in planktonic culture and early-stage (6 h) biofilms, while levels of resistance are maintained in mature biofilms^41^. Therefore, we propose that it is more likely the increased azole resistance phenotype of 5% CO_2_ biofilms displayed is contributed to via another mechanism, possibly increased ECM deposition. β-1,3-Glucan, a major component of biofilm ECM, can bind to azole antifungals and sequester them to prevent passage to the cells^49^. We have observed that, after 48 h, 5% CO_2_ biofilms, while containing similar cell numbers to 0.03% CO_2_ biofilms, were significantly thicker. This could suggest more ECM material being produced in high CO_2_ environments, contributing to the increased azole resistance. Furthermore, Miconazole treatment has been shown to generate superoxide radicals within *C. albicans* biofilms and leads to the increased expression of *SOD5* and *SOD6* (encode superoxide dismutase enzymes) in an attempt to protect against the toxic superoxides. A *sod4Δ/Δsod5Δ/Δsod6Δ/Δ* triple mutant is hypersensitive to Miconazole treatment when growing as a biofilm^50^. Our transcriptome analysis revealed that *SOD6* and *SOD4* were upregulated in 5% CO_2_ biofilms, providing a potential further mechanism for increased Miconazole resistance.

Our study identified that TFs involved in iron homoeostasis are important for *C. albicans* biofilm growth. Principal among these were the Hap TFs, which come together to form the HAP complex, a CCAAT box-binding transcriptional regulator, under iron-limiting conditions. Genetic studies have revealed a requirement of *HAP2*, *HAP3*, *HAP5* and *HAP43* for growth in low-iron media^30,51^. Thus, the biofilm formation defect exhibited by the *hap2Δ/Δ*, *hap3Δ/Δ*, *hap5Δ/Δ* and *hap43Δ/Δ* mutants in 0.03% CO_2_ could be explained by this growth deficiency since RPMI-1640 media has a low-iron content. Nevertheless, this makes it even more intriguing that simply an increase in ambient CO_2_ levels was able to significantly increase the biofilm growth of these mutants. The HAP complex represses a GATA-type TF called Sfu1; Sfu1 is responsible for repressing iron-uptake genes along with *SEF1* under iron-replete conditions. Sef1 activates iron-uptake genes as well as *HAP43*, *HAP2* and *HAP3*^52^, in this way the HAP complex is able to induce iron-uptake pathways while repressing iron-utilisation genes^31,52^. Deletion of Sef1 results in the aberrant downregulation of all the major iron-uptake pathways of *C. albicans* in low-iron conditions^52^. Due to the fact the *sef1Δ/Δ* and *ssn3Δ/Δ* mutants had defective biofilm growth in both CO_2_ conditions, we hypothesised that a high CO_2_ environment may influence one or both of these directly, causing the HAP complex to become at least partially redundant under these conditions. We did see a number of genes involved in iron acquisition had increased transcript levels in 48 h 5% CO_2_ biofilms. For example, *FTR2*, which encodes the high-affinity iron permease Ftr2, had increased expression. In addition, *CFL4*, which encodes a putative ferric reductase, is regulated by Sef1 and induced in low-iron conditions^52^ was also upregulated. Finally, the *CSA2* and *RBT5* genes, which encode proteins involved in the acquisition of iron from haem groups, were also upregulated. Csa2 and Rbt5 have both previously been found to be required for normal biofilm formation in RPMI-1640 media^53,54^.

The increased Sef1 abundance in mature wild-type biofilms under high CO_2_, combined with no such increase in the *ssn3* null mutant, implies that this was an Ssn3-dependent process and argues against CO_2_ acting on Sef1 directly. Moreover, Sef1 levels in the *ssn3Δ/Δ* background were extremely low in both CO_2_ environments, consistent with the identical biofilm formation defect of the *sef1Δ/Δ* and *ssn3Δ/Δ* mutants. Conversely, Sef1 levels were high in the *sfu1Δ/Δ* background. Given the importance of Sef1 for biofilm formation on a silicone surface in our assay, this could explain the significantly increased biofilms formed by the *sfu1Δ/Δ* mutant. These cells are likely primed for enhanced iron acquisition right from the beginning of biofilm growth, increasing their fitness in this scenario. Ssn3 is a cyclin-dependent kinase and functions in a post-translational control loop of Sef1 with Sfu1^32^. We did not observe a significant effect on *SEF1* mRNA levels within 5% CO_2_ biofilms after 48 h. Thus, the increase in Sef1 abundance observed in 5% CO_2_ biofilms is likely due to CO_2_ acting at the post-translational level and our data are consistent with Ssn3 playing a role in this. This is quite surprising since Ssn3 has been found to be dephosphorylated and hence inactive in planktonic cells grown in 5% CO_2_^55^. We see that a correctly functioning iron homoeostatic network is required for the CO_2_-mediated increase in *C. albicans* attachment to a silicone surface and it has previously been shown that the adhesin Als3 plays a direct role in iron acquisition from ferritin^56^, demonstrating an association between these processes. It should be noted that, besides their key roles in iron homoeostasis, the HAP complex and Ssn3 have numerous and varied tasks throughout the cell^31,57^. Therefore, although our data points towards the iron homoeostatic roles being crucial for *C. albicans* biofilm growth, other processes cannot be ruled out.

The increased azole resistance of *C. albicans* biofilms in 5% CO_2_ along with the observed iron-starvation tolerance when treated with the Fe^3+^ chelator Deferasirox has important implications for the development of potential biofilm treatment strategies. At a clinical level, this also indicates the location of a *C. albicans* biofilm within the body should be taken into consideration when deciding upon the most effective treatment. Encouragingly, 2-DG was able to attenuate *C. albicans* biofilm growth in both 5% and atmospheric (0.03%) CO_2_ environments. 2-DG has exhibited antimicrobial effects against fungal moulds^58^ and bacterial biofilms^59^. This, together with its action against *C. albicans* biofilms presented here, highlights the potential for 2-DG to be used an anti-biofilm therapeutic. It may be particularly useful for medical devices such as VPs, which are situated in CO_2_-rich environments in the body and are often colonised by a mixture of bacterial and fungal species^60–63^. It is important to note, however, that 2-DG was unable to eradicate *C. albicans* biofilm growth completely. It may be that 2-DG treatment is beneficial in combination with other compounds, such as iron chelators or traditional antifungals; this possibility is yet to be explored.

In conclusion, our data demonstrate that elevated levels of CO_2_ act as an important driver of *C. albicans* biofilm formation, growth and maturation. These CO_2_-mediated effects are not only likely to have important medical ramifications, particularly in the context of prosthetics and airway management devices, but also for host infections in CO_2_-rich environments in the body.

## Methods

### *Candida* strains and growth media

*Candida* strains (Supplementary Table S1) were routinely grown at 30 °C in yeast peptone dextrose (YPD) media (2% peptone (BD Bacto), 2% d-glucose (Fisher Scientific), 1% yeast extract (BD Bacto)). For biofilm growth assays, *Candida* biofilms were grown at 37 °C in RPMI-1640 media (Sigma-Aldrich, R8755) supplemented with 80 µg/ml uridine (Sigma-Aldrich, U3750) if required. The *C. albicans* TFKO library we used was generated by Homann et al. as described in ref. ^30^. Briefly, deletions were constructed using auxotrophic marker cassettes targeted with long-flanking homology. *C. albicans* strain SN152 (*arg4Δ/Δ*, *leu2Δ/Δ*, *his1Δ/Δ*, *URA3/ura3Δ*, *IRO1/iro1Δ*) was transformed with *C. maltosa LEU2* and *Candida dubliniensis HIS1*, which integrated at the targeted transcriptional regulator locus, deleting both alleles. A ‘wild-type’ control strain (SN250) was created by the reintroduction of single alleles of *C. dubliniensis HIS1* and *Candida maltosa LEU2* into SN152^30^. The *ssn3Δ/Δ* null mutant was constructed using the same method^64^. The CDH107 (*ras1Δ/Δ*), CR276 (*cdc35Δ/Δ*) and WYF2 (*CDC35*^*ΔRA*^) strains were constructed as described in refs. ^65–67^, respectively.

### In vitro biofilm growth assays

*C. albicans* biofilms were grown on a polydimethylsiloxane (PDMS) silicone elastomer (Provincial Rubber, S1). The silicone was cut into 1 cm^2^ squares and placed in clips in a modified 24-well plate lid (Academic Centre for Dentistry Amsterdam, AAA-model) so they could be suspended in media within a 24-well plate. Silicone squares were incubated in 1 ml 50% Donor Bovine Serum (DBS; Gibco, 16030074) for 30 min at 30 °C, then washed twice with 1 ml phosphate-buffered saline (PBS) to remove excess DBS. In all, 1 ml of *C. albicans* were added to each well of a 24 well plate (Greiner Bio-one, CELLSTAR, 662160) following resuspension in PBS at an OD_600_ of 1.0 and the lid with the silicone squares attached was placed on top so the silicone squares protrude into the cell suspension. Plates were then incubated at 37 °C (in either 0.03% CO_2_ or 5% CO_2_) without shaking for 90 min to allow cell attachment to the silicone. After the attachment phase, the silicone squares were washed twice with 1 ml PBS to remove any unattached cells and transferred to 1 ml RPMI-1640 media (Sigma-Aldrich, R8755). They were then incubated at 37 °C (in either 0.03% CO_2_ or 5% CO_2_) without shaking for 48 h to allow biofilm maturation.

### Biofilm quantification via XTT assay

Biofilm growth was quantified using an XTT assay^29^. Biofilms were washed twice with 1 ml PBS to remove any planktonic cells before proceeding to quantification. After washing, the biofilms were transferred to a new pre-sterilised 24-well plate containing 30 µg/ml XTT labelling reagent (Roche, 11465015001) and incubated at 37 °C for 4 h. After incubation, the biofilms were removed from the 24-well plate and the absorbance of the remaining XTT labelling reagent was measured at 492 nm using a BMG LABTECH FLUOstar Omega plate reader.

### Biofilm quantification via dry biomass assay

Biofilms were set up and grown on a 30 cm^2^ square of PDMS silicone elastomer (Provincial Rubber, S1), which was attached to the base of a petri dish. Silicone squares were incubated in 30 ml 50% DBS for 30 min at 30 °C, then washed with 30 ml PBS to remove excess DBS. In all, 30 ml *C. albicans* were added to each petri dish following resuspension in PBS at an OD_600_ of 0.1. Plates were then incubated at 37 °C (in either 0.03% CO_2_ or 5% CO_2_) without shaking for 90 min to allow cell attachment to the silicone. After the attachment phase, the silicone squares were washed with PBS to remove any unattached cells, then bathed in 30 ml RPMI-1640 media. They were then incubated at 37 °C (in either 0.03% CO_2_ or 5% CO_2_) without shaking for 24 or 48 h to allow biofilm maturation. Following maturation, biofilms were washed with PBS to remove any planktonic cells and harvested using 2 cm blade cell scrapers (Jet Biofil, CSC011025) to scrape adhered biofilms into PBS. The resultant biofilm suspension was collected and passed through a pre-weighed 47 mm diameter 0.45 µm nitrocellulose membrane filter (Whatman, 7184 004) by vacuum filtration. The membranes were allowed to air dry and weighed on a 0.1 mg microbalance (Ohaus Explorer Pro, EP64C).

### *C. albicans* TFKO screen

*C. albicans* mutants from a TFKO library were screened for biofilm-forming ability in both 0.03 and 5% CO_2_. Biofilms were seeded and grown for 48 h on a PDMS silicone elastomer (Provincial Rubber, S1) as described above. Biofilm growth was quantified using the XTT assay. Experiments were performed in biological triplicate.

### Iron starvation of *C. albicans* biofilms

Biofilms were set up as described in the ‘In vitro biofilm growth assay’ section above except they were incubated at 37 °C in either 0.03% CO_2_ or 5% CO_2_ for 48 h in RPMI-1640 containing varying concentrations of the Fe^2+^ chelator 3-(2-Pyridyl)-5,6-diphenyl-1,2,4-triazine-p,p’-disulfonic acid monosodium salt hydrate (Ferrozine—Sigma-Aldrich, 160601) or the Fe^3+^ chelator Deferasirox (Cambridge Bioscience, CAY16753-5). Ferrozine was made as a 100 mM stock solution in sterile MQ H_2_O and diluted in RPMI-1640 to final concentrations of 250–500 μM. Deferasirox was made as a 20 mg/ml stock solution in dimethyl sulfoxide (DMSO) and diluted in RPMI-1640 to final concentrations of 70–210 µg/ml. Relevant solvent controls were included. Final biofilms were quantified using an XTT assay.

### Preparation of PDMS-coated microscope slides

To prepare PDMS for coating microscope slides 16 g (6.16 × 10^−4^ mol) silanol-terminated PDMS (cSt 1000, M_W_ 26000, from Fluorochem Ltd.) and 0.26 g (1.248 mmol, 1:4 stoichiometric ratio) cross-linking agent tetraethyl orthosilicate (Sigma-Aldrich, 131903) were mixed at 3500 r.p.m. for 1 min using a DAC 150FV2-K speedmixer. At this point, 720 µl tin(II) ethylhexanoate (Sigma-Aldrich, S3252) made up at a concentration of 0.6 M in toluene was added as a catalyst and the mix spun for a further 60 s at 3500 r.p.m. The elastomer mixture was then doctor bladed onto microscope slides using an automatic precision film applicator MTCX4 (Mtv-Messtechnik—blade width = 70 mm, thickness adjustability 0–3000 µm). The doctor blade height was set 10 µm higher than the thickness of the microscope slide. The elastomer mix was poured over the top of the slide (with a bias towards the side of the microscope slide closest to the doctor blade), and then the doctor blade is moved at a constant speed over the substrate. The microscope slide was then air cured for 2 h before being heat cured for 18 h in a 70 °C oven.

### Preparation of *C. albicans* biofilms on silicone-coated slides for microscopy

Biofilms were grown directly on microscope slides that had been precoated with a PDMS silicone polymer (see above) for confocal imaging. Biofilms were grown with a prefabricated well. PDMS-coated microscope slides were incubated with 400 µl 50% DBS (Gibco, 16030074) in the wells for 30 min at 30 °C and washed twice with 400 µl PBS. *C. albicans* overnight cultures were grown in YPD at 30 °C and washed twice in PBS. In all, 400 µl of the OD_600_ 1.0 standard cell suspension was added to the wells and incubated at 37 °C (in either 0.03% CO_2_ or 5% CO_2_) without shaking for 90 min to allow cell attachment to the silicone surface. After attachment, the microscope slides were washed twice with 400 µl PBS to remove any unattached cells and then incubated at 37 °C with 400 µl RPMI-1640 media in the wells for 6, 24 or 48 h (in either 0.03% CO_2_ or 5% CO_2_). Biofilms were washed twice with 400 µl PBS and then incubated in the dark for 45 min at 30 °C in 400 µl PBS containing 50 µg/ml ConA-FITC (Sigma-Aldrich, C7642) and 20 µM FUN-1 (Invitrogen Molecular Probes, F7030). After incubation with the dyes, the stained biofilms were washed again with 400 µl PBS to remove any residual dye. The well was removed and two drops of ProLong^TM^ Diamond Antifade Mountant (Invitrogen, P36965) was added to each stained biofilm. A cover slip was placed on top and the microscope slides were incubated in the dark at room temperature overnight to allow the mountant to cure.

### Preparation of *C. albicans* biofilms on chamber slides for microscopy

Biofilms were grown within Ibidi µ-slide eight-well microscope slides with polymer coverslip bottom (Ibidi, 80821) following the procedure described above with some alterations. Specifically, DBS was not used as the chamber slides had been precoated with poly-L-lysine to aid attachment. These biofilms were stained with 400 µl FilmTracer SPYRO Ruby biofilm matrix stain (Invitrogen Molecular Probes, F10318). Coverslips were not added as the bottom of the chamber slide is a coverslip.

### CSLM of *C. albicans* biofilms

Stained biofilms grown on silicone-coated slides or Ibidi chamber slides were imaged using a Zeiss LSM880/Elyra/Axio Observer.Z1 Confocal Microscope (Carl Zeiss Inc.) using the 488 nm argon and the 561 nm DP55 lasers. Images of the green (ConA-FITC) and the red (FUN-1) fluorescence were taken simultaneously using a multitrack mode. Images of the SPYRO Ruby biofilm matrix stain were taken individually using a single track. *Z*-stacks were taken using the inbuilt ‘optimal’ settings to determine the optimal intervals (typically 1.5–2.0 µm slices) based on sample thickness and magnification. The ×20 and oil-immersion ×40 objective lenses were used throughout. The image acquisition software used was ZENBlack and the image processing software was ZENBlue.

### RNA isolation from *C. albicans* biofilms

Total RNA was extracted in biological triplicate per condition (0.03% and 5% CO_2_) using the E.Z.N.A.^TM^ Yeast RNA Kit (Omega Bio-Tek, R6870-01) as per the manufacturer’s instructions with a few modifications. Specifically, *C. albicans* CAI-4 biofilms were seeded and grown in 0.03 and 5% CO_2_ as described for in vitro biofilm growth assays. Mature biofilms were washed twice with 1 ml ice-cold PBS to remove any planktonic cells. Biofilm cells were harvested by transferring silicone squares upon which the biofilms were growing into 5 ml cold SE buffer/2-mercaptoethanol (provided in the E.Z.N.A.^TM^ Yeast RNA Kit) and vortexing at 2500 r.p.m. for 1 min. The resulting biofilm cell suspension was pelleted by centrifugation at 4000 r.p.m. for 10 min at 4 °C. The supernatant was discarded and the cells were re-suspended in fresh 1 ml cold SE buffer/2-mercaptoethanol; this cell suspension was transferred to a 2 ml Eppendorf tube. The cell suspension was centrifuged again for 10 min at 4 °C, the supernatant was discarded and the pellet was re-suspended in 480 µl fresh SE buffer/2-mercaptoethanol. The biofilm cell suspension was incubated with 80 µl lyticase stock solution (5000 units/ml in SE buffer) at 30 °C for 90 min. The resulting spheroplasts were pelleted by centrifugation at 2900 r.p.m. for 10 min at 4 °C and the supernatant was aspirated and discarded. The spheroplasts were gently re-suspended in 350 µl YRL buffer/2-mercaptoethanol (provided in the E.Z.N.A.^TM^ Yeast RNA Kit). The rest of the RNA extraction proceeded as per the manufacturer’s instructions including the optional DNase digestion step. RNA was eluted in 30 µl DEPC water; the concentration and purity established using a NanoDrop ND-1000 spectrophotometer (NanoDrop Technologies) and stored at −80 °C.

### Library preparation and RNA sequencing

RNA samples were sent to the Centre for Genome Enabled Biology and Medicine (Aberdeen, UK) for library preparation and sequencing. Before library preparation, the quality and quantification of RNA samples were evaluated with TapeStation (Agilent) and Qubit (Thermal Fisher). Samples with a minimum RNA Integrity Number of 8.0 proceeded to library preparation. The input of RNA was based on the specifically measured RNA concentration by Qubit. The mRNA-Seq libraries were prepared using the TruSeq™ Stranded mRNA Sample Preparation Kit (Illumina) according to the manufacturer’s instructions. Briefly, Poly-A RNA were purified from 500 ng of total RNA with 1 µl (1:100) ERCC spike (Thermal Fisher) as an internal control using RNA purification oligo(dT) beads, fragmented and retrotranscribed using random primers. Complementary DNAs were end-repaired, and 3-adenylated, indexed adaptors were then ligated. Fifteen cycles of PCR amplification were performed, and the PCR products were cleaned up with AMPure beads (Beckman Coulter). Libraries were validated for quality on the Agilent DNA1000 Kit and quantified with the qPCR NGS Library Quantification Kit (Roche). The final libraries were equimolar pooled and sequenced using the High Output 1×75 Kit on the Illumina NextSeq500 platform producing 75 bp single-end reads. For each library, a depth of 50–70 M reads was generated.

### Analysis of RNA-Seq data

Analysis of RNA-Seq data was performed using the Galaxy web platform^68^. The quality of the RNA sequencing reads was checked using FastQC v0.11.5^69^ with default settings. Low-quality ends (Phred score <20) and any adaptor sequences were trimmed using TrimGalore! v0.4.3^70^. Reads that became <40 bp after trimming were removed from further analysis. After trimming, 97.7% of initial reads remained and the quality was checked again using FastQC v0.11.5^69^. There were no Poly-A reads (>90% of the bases equal A), ambiguous reads (containing N) or low-quality reads (>50% of the bases with a Phred score <25). After processing, the mean Phred score per read was 35. Processed reads were aligned with the reference *C. albicans* genome SC5314 version A21-s02-m09-r10 using HISAT2 v2.1.0^71^ with single-end reads and reverse strand settings (rest of the settings were default). After alignment, the number of mapped reads that overlapped CDS features in the genome (using the *C. albicans_SC5314_version_A21-s02-m09-r10_features.gtf* annotation file^72^) were determined using htseq-count v0.9.1^73^ with default settings. Reads aligning to multiple positions or overlapping more than one gene were discarded, counting only reads mapping unambiguously to a single gene. Differential gene expression analysis between conditions (0.03 and 5% CO_2_) was performed using DESeq2 v1.18.1^74^ with default settings. Raw and normalised data files were deposited with the Gene Expression Omnibus repository (GSE172004).

### GSEA of transcription profiles

Downstream analysis of RNA-Seq data was performed using the PreRanked tool of GSEA (Broad Institute)^33^, which compares a pre-ranked significantly differentially expressed gene list to a functional gene set database. FDR *q* values were calculated based on 1000 permutations. The gene set database used was assembled by Sellam et al. as described in ref. ^75^, which is based on experimental analyses from published studies, GO term categories curated by the *Candida* Genome Database^76^ and protein–protein interaction information derived from *Saccharomyces cerevisiae* data curated by the *Saccharomyces* Genome Database^77^. Gene set networks were generated in Cytoscape 3.7.1 (Available at: https://cytoscape.org/)^34^ using the EnrichmentMap plug-in (available at http://apps.cytoscape.org/apps/enrichmentmap). Gene expression heat maps based on GO term categories were created using the Pheatmap package in R Studio.

### Antifungal treatment of *C. albicans* biofilms

Biofilms were set up as described in the ‘In vitro biofilm growth assay’ section above and grown in RPMI-1640 for 24 h at 37 °C. The biofilms were then transferred to fresh RPMI-1640 media containing a select antifungal. Four antifungals were tested; Fluconazole, Miconazole, Nystatin, and Caspofungin. Fluconazole (Santa Cruz Biotechnology, sc-205698) was made as a 50 mg/ml stock solution in ethanol and diluted in RPMI-1640 to final concentrations ranging from 8 to 256 µg/ml. Miconazole (Santa Cruz Biotechnology, sc-205753) was made as a 50 mg/ml stock solution in DMSO and also diluted in RPMI-1640 to final concentrations ranging from 8 to 256 µg/ml. Nystatin (Santa Cruz Biotechnology, sc-212431) was made as a 5 mg/ml stock solution in DMSO and diluted in RPMI-1640 to final concentrations ranging from 1 to 8 µg/ml. Caspofungin (Sigma SML0425) was made as a 16 mg/ml stock solution in DMSO and diluted in RPMI-1640 to final concentrations ranging from 0.03125 to 1 µg/ml. Drug vehicle controls (0.5% ethanol for Fluconazole, 0.5% DMSO for Miconazole and Nystatin, 0.1% DMSO for Caspofungin) were used in all cases. The biofilms matured in the RPMI-1640 media containing the select antifungal for a further 24 h at 37 °C in both 0.03 and 5% CO_2_ before proceeding to quantification via the XTT assay. Experiments were performed in biological and technical triplicate.

### *C. albicans* attachment assay

CAI-4 cells were seeded onto silicone-coated microscope slide for 90 min at an OD_600_ of 0.1 without DBS pre-treatment. The slide surface was washed twice with 400 µl PBS to remove any unattached cells. Images were taken using a Leica DMR fitted with a Leica DFC9000 GT camera using a ×20 objective lens and brightfield settings. The image acquisition software used was the Leica Application Suite X package. Using identical microscope settings throughout, five images were taken of each of three biological replicates in both 0.03 and 5% CO_2_, and the cells per image were counted using the ‘Cell Counter’ function in ImageJ.

### 2-DG treatment of *C. albicans* biofilms

Biofilms were set up as described in the ‘In vitro biofilm growth assay’ section above except that they were incubated at 37 °C in either 0.03% CO_2_ or 5% CO_2_ for 48 h in RPMI-1640 containing varying concentrations of 2-DG. 2-DG (Sigma-Aldrich, D6134) was made up as a 20% stock solution in sterile MQ H_2_O and diluted in RPMI-1640 to final concentrations of 0.25–1%. Biofilms were quantified using XTT assays and images were also taken. Experiments were performed in biological and technical triplicate.

### *C. albicans* biofilm dispersion assay

Biofilms were set up as described in the ‘In vitro biofilm growth assay’ section above and grown in RPMI-1640 for 48 h at 37 °C. The spent media, containing dispersed cells, was sonicated at amplitude 4 µm for 10 s to separate clumps of hyphal cells (previous work in our laboratory has demonstrated that these sonication settings do not affect viability). After sonication, the dispersed cells were diluted 1 in 10 and 200 µl of this suspension was plated on YPD agar plates in triplicate. YPD plates were incubated for 48 h at 37 °C to allow colonies to form, at which point the number of colonies were manually counted using a Stuart Scientific Colony Counter. Experiments were performed in biological and technical triplicate.

### Cell wall stress sensitivity spotting assay

CAI4 cells were grown overnight in 5 ml YPD, then diluted to an OD_600_ of 1. A dilution series was created (10^0^, 10^−1^, 10^−2^, 10^−3^, 10^−4^, 10^−5^) and spotted onto YPD agar plates containing 50 µg/ml Congo Red (Sigma-Aldrich, C6277) and YPD agar plates containing 25 µg/ml Calcofluor White (Sigma-Aldrich, 18909). The plates were incubated for 48 h at 30 °C.

### Cell wall stress sensitivity planktonic growth assay

CAI4 cells were grown overnight in 5 ml YPD, then diluted to an OD_600_ of 0.1 in YPD containing increasing concentrations of Congo Red (3.125-50 µg/ml) or Calcofluor White (1.5625-25 µg/ml) in a 96-well plate (Greiner Bio-one, CELLSTAR, 655180). The plates were incubated with 400 r.p.m. double orbital shaking in a BMG LABTECH SPECTROstar^Nano^ plate reader for 48 h at 37 °C in both 0.03 and 5% CO_2_. Growth was measured by OD_600_ readings taken every 10 min. The lag times and growth rates for each condition were calculated by fitting the growth curves to a Baranyi model using the DMFit software.

### Protein extraction from *C. albicans* biofilms

Biofilms were seeded on the bottom of wells in a 6-well plate (Greiner Bio-one, CELLSTAR, 657160) and grown in RPMI-1640 for 48 h at 37 °C. Mature biofilms were washed twice with 1 ml ice-cold PBS to remove any planktonic cells. In all, 1 ml ice-cold lysis buffer (100 mM sodium phosphate buffer pH 7.8, 5 mM EDTA, 0.1% Triton, 50 mM NaCl, Pierce protease and phosphatase inhibitor (Thermo Scientific, A32961)) was added to each well and biofilm cells were scraped from the bottom of the wells. Three biofilms worth of material per strain and condition were combined and centrifuged at 4000 rpm for 5 min at 4 °C. The supernatant was discarded and biofilm cells resuspended in 500 µl lysis buffer. This cell suspension was added to a pestle and mortar on dry ice and frozen with liquid nitrogen; cells were mechanically lysed by grinding. Cell lysates were collected and centrifuged at 14,000 r.p.m. for 5 min at 4 °C. Protein concentration of the supernatants was measured by the Bradford assay. Proteins were concentrated by trichloroacetic acid precipitation.

### Immunoblotting

Protein extracts (10 µg total protein per sample) were analysed by sodium dodecyl sulphate–polyacrylamide gel electrophoresis (Wako, SuperSep Ace 199-14971) and immunoblotted with a 9E10 anti-c-myc antibody (Sigma-Aldrich, M4439) for Myc-tagged Sef1. Immunoblots were also probed with an anti-PGK1 antibody (M. F. Tuite laboratory) as a loading control. All western blots derive from the same experiment and were processed in parallel.

### Statistical analyses

Statistically significant differences between dependent variables were assessed with either a paired two-tailed *t* test or, more often, with a two-way analysis of variance followed by Tukey test for multiple comparisons. All statistical analyses were carried out using GraphPad Prism, version 9.0.2.

### Reporting summary

Further information on research design is available in the Nature Research Reporting Summary linked to this article.

## Supplementary information

Supplementary Information

Reporting Summary

## Data Availability

Source data are provided with this paper for the RNA sequencing experiments within this study, within the Gene Expression Omnibus (GEO) repository with the accession code GSE172004. All other data that support the findings of this study are available from the corresponding author upon reasonable request.
