# Supplementary material for: CO2 enhances the formation, nutrient scavenging and drug resistance properties of C. albicans biofilms
Source: NPJ Biofilms Microbiomes. 2021 Aug 12;7:67. doi: 10.1038/s41522-021-00238-z (PMC8361082; doi:10.1038/s41522-021-00238-z)

Supplementary Table S1

Strain	Species	Genotype/Parent	Source/Reference
SN152	*C. albicans*	*ura3*::*URA3/ura3*::imm434 *iro1::IRO1/iro1*::imm434 *his*1::hisG/*his1*::hisG *leu2/leu2* *arg4/arg4*	Homann, O. R., *et. al.* (2009)
SN250 (TFKO Library Reference Strain)	*C. albicans*	*ura3*::*URA3/ura3*::imm434 *iro1::IRO1/iro1*::imm434 *his*1::hisG/*his1*::hisG *leu2*::*CmLEU2/leu2*::*CdHIS1* *arg4/arg4*	Homann, O. R., *et. al.* (2009)
CAI4	*C. albicans*	*ura3*::imm434/*ura3*::imm434 *iro1/iro1*::imm434	Mühlschlegel Lab
CAI4pSM2	*C. albicans*	CAI4 transformed with pSM2, *URA3* integrating plasmid	Mühlschlegel Lab
TFKO Library	*C. albicans*	SN152	Homann, O. R., *et. al.* (2009)
ssn3Δ/Δ	*C. albicans*	SN152	Noble, S. M., *et. al.* (2010)
SN423 (Sef1-Myc, Wild Type)	*C. albicans*	*SEF1-13xMyc/SEF1* *ura3*::*URA3/ura3*::imm434 *iro1::IRO1/iro1*::imm434 *his*1::hisG/*his1*::hisG *leu2::CmLEU2/leu2::CdHIS1* *arg4/arg4*	Chen, C., *et. al.* (2012)
SN702 (Sef1-Myc, *sfu1Δ/Δ*)	*C. albicans*	*SEF1-13xMyc/SEF1* *ura3*::*URA3/ura3*::imm434 *iro1::IRO1/iro1*::imm434 *his*1::hisG/*his1*::hisG *leu2/leu2* *sfu1::CmLEU2/sfu1::CdHIS1* *arg4/arg4*	Chen, C., *et. al.* (2012)
SN908 (Sef1-Myc, *ssn3Δ/Δ*)	*C. albicans*	*SEF1-13xMyc/SEF1* *ura3*::*URA3/ura3*::imm434 *iro1::IRO1/iro1*::imm434 *his*1::hisG/*his1*::hisG *leu2/leu2* *ssn3::CmLEU2/ssn3::CdHIS1* *arg4/arg4*	Chen, C., *et. al.* (2012)
CDH107 (*ras1Δ/Δ*)	*C. albicans*	CAI4	Klengel, T. *et. al.* (2005)
CR276 (*cdc35Δ/Δ*)	*C. albicans*	CAI4	Rocha, C. R. *et. al.* (2001)
WYF2 (*CDC35*$^{\Delta RA}$)	*C. albicans*	CR276 transformed with pClp-*cdc35*$^{\Delta RA}$	Fang, H. M. *et. al.* (2006)
tpk1Δ/Δ	*C. albicans*	CAI4	Mühlschlegel Lab
tpk2Δ/Δ	*C. albicans*	CAI4	Mühlschlegel Lab
G-3065	*C. albicans*	Clinical Isolate	Failed Voice Prosthesis
G-8424	*C. albicans*	Clinical Isolate	Failed Voice Prosthesis
G-1625	*C. albicans*	Clinical Isolate	Failed Voice Prosthesis

Supplementary Table S1: *Candida albicans* strains used in this study.

Supplementary Table S2

TFKO Mutant	Biofilm Growth	
	0.03% CO_2	5% CO_2
tup1Δ/Δ	– – – ($p < 0.001$)	– – – ($p < 0.001$)
sef1Δ/Δ	– – ($p = 0.002$)	– – ($p < 0.001$)
swi4Δ/Δ	– – ($p < 0.001$)	– – ($p = 0.002$)
pho4Δ/Δ	– – – ($p < 0.001$)	– ($p = 0.049$)
bcr1Δ/Δ	– – – ($p < 0.001$)	– – ($p = 0.009$)
efg1Δ/Δ	– – ($p = 0.007$)	– – – ($p = 0.044$)
hap2Δ/Δ	– – – ($p < 0.001$)	n.s. ($p = 0.561$)
rbf1Δ/Δ	– – ($p < 0.001$)	n.s. ($p = 0.873$)
rob1Δ/Δ	– – ($p < 0.001$)	n.s. ($p = 0.477$)
fgr15Δ/Δ	– – ($p = 0.005$)	n.s. ($p = 0.637$)
dal81Δ/Δ	– – ($p = 0.007$)	n.s. ($p = 0.682$)
mig1Δ/Δ	– – ($p = 0.014$)	n.s. ($p = 0.264$)
brg1Δ/Δ	– – ($p = 0.022$)	n.s. ($p = 0.998$)
C4_00260WΔ/Δ	– – ($p = 0.023$)	n.s. ($p = 0.915$)
zcf27Δ/Δ	– – ($p = 0.047$)	n.s. ($p = 0.870$)
C1_13880CΔ/Δ	– ($p = 0.009$)	n.s. ($p = 0.803$)
crz1Δ/Δ	– ($p = 0.022$)	n.s. ($p = 0.474$)
hap43Δ/Δ	– ($p = 0.030$)	n.s. ($p = 0.338$)
leu3Δ/Δ	n.s. ($p = 0.120$)	– – ($p < 0.001$)
mbp1Δ/Δ	n.s. ($p = 0.085$)	– – ($p = 0.007$)
bas1Δ/Δ	n.s. ($p = 0.563$)	– ($p = 0.016$)
try6Δ/Δ	n.s. ($p = 1.000$)	– ($p = 0.037$)
mac1Δ/Δ	++ ($p = 0.020$)	n.s. ($p = 0.847$)
zcf30Δ/Δ	++ ($p = 0.038$)	n.s. ($p = 0.640$)
zcf17Δ/Δ	+ ($p = 0.023$)	n.s. ($p = 0.735$)

Supplementary Table S2: Summary of the transcription factor knockout (TFKO) mutants which had significantly altered biofilm growth in 0.03% and/or 5% CO_2 environments within our screen. Biofilms were quantified via XTT assays and normalised to the wild type XTT readout within each CO_2 environment; – – – <40% XTT activity relative to wild type, – – 40-70%, – >70%, + 110-130%, ++ >130%, n.s. non-significant. Green indicates transcription factors previously known to have a role in biofilm regulation as per Gene Ontology analysis. Grey indicates transcription factors involved in maintaining cellular iron homeostasis.

Supplementary Figure S1

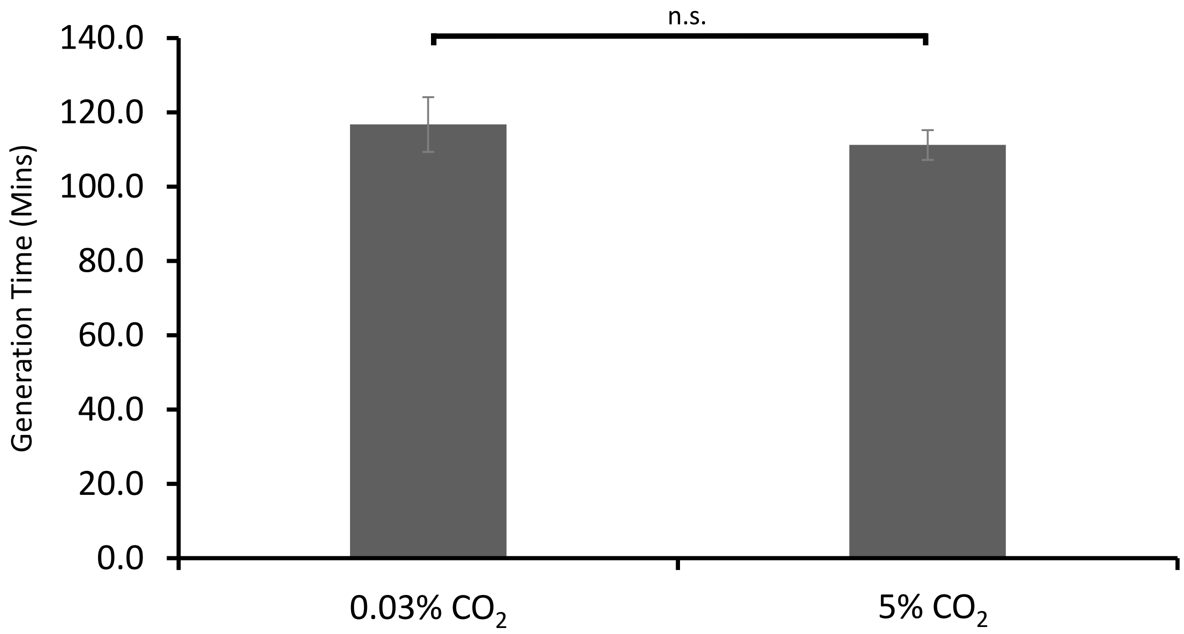

Supplementary Figure S1: Planktonic growth of *C. albicans* CAI-4 in RPMI-1640 in 0.03% and 5% CO_2. Overnight cultures were inoculated to OD_{600} 0.1 in RPMI-1640 in 24-well plates. They were grown for 48 hours with 400rpm double orbital shaking in a BMG LABTECH SPECTROstar^{Nano} plate reader for 0.03% CO_2 conditions and a BMG LABTECH CLARIOstar plate reader for 5% CO_2 conditions. Growth was measured by OD_{600} readings taken every 10 mins. Graph represents three biological replicates each containing technical triplicate. A paired two-tail t test was carried out; n.s. = not significant.

Supplementary Figure S2

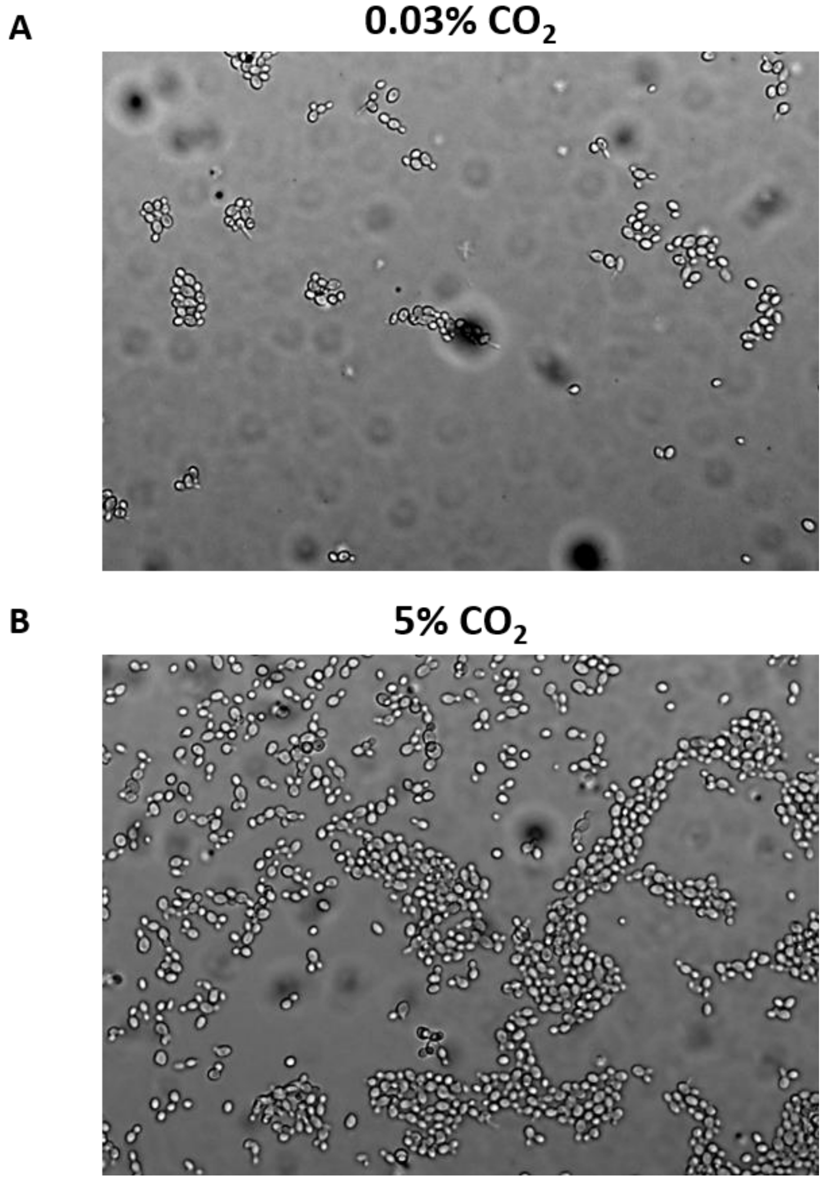

Supplementary Figure S2: Attachment of *C. albicans* CAI4 cells to a silicone surface in 0.03% and 5% CO_2. *C. albicans* CAI4 cells were seeded for 90 mins onto silicone-coated microscope slides in 0.03% CO_2 or 5% CO_2, unattached cells were washed off and images taken at 20x objective magnification. Experiment was repeated in triplicate and representative images are shown. **(A)** Representative image of a slide section from 0.03% CO_2. **(B)** Representative image of a slide section from 5% CO_2.

Supplementary Figure S3

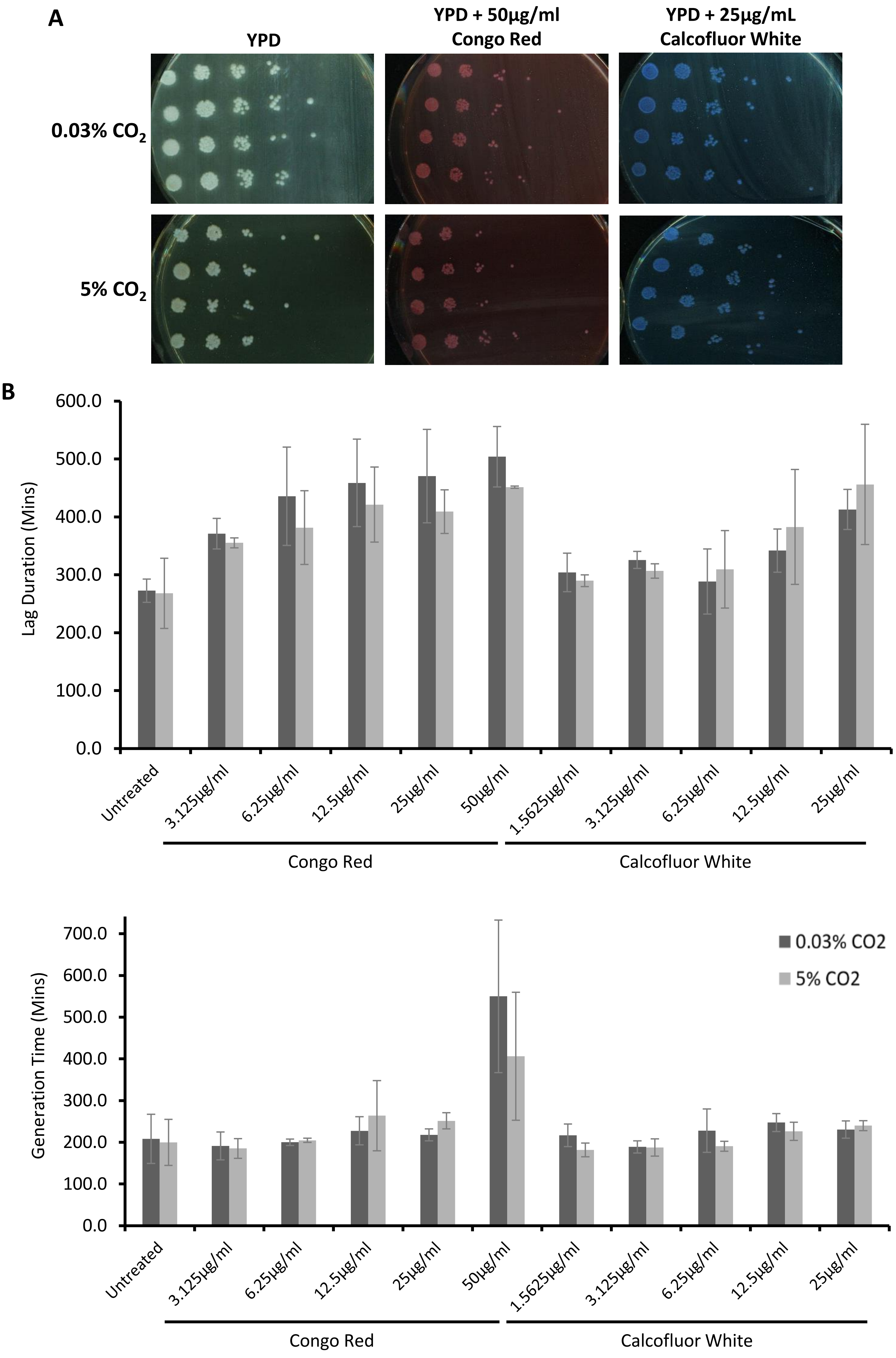

Suppleme
5% CO_2.
assays wi
of the pl
technical
tests for r

Supplementary Figure S4

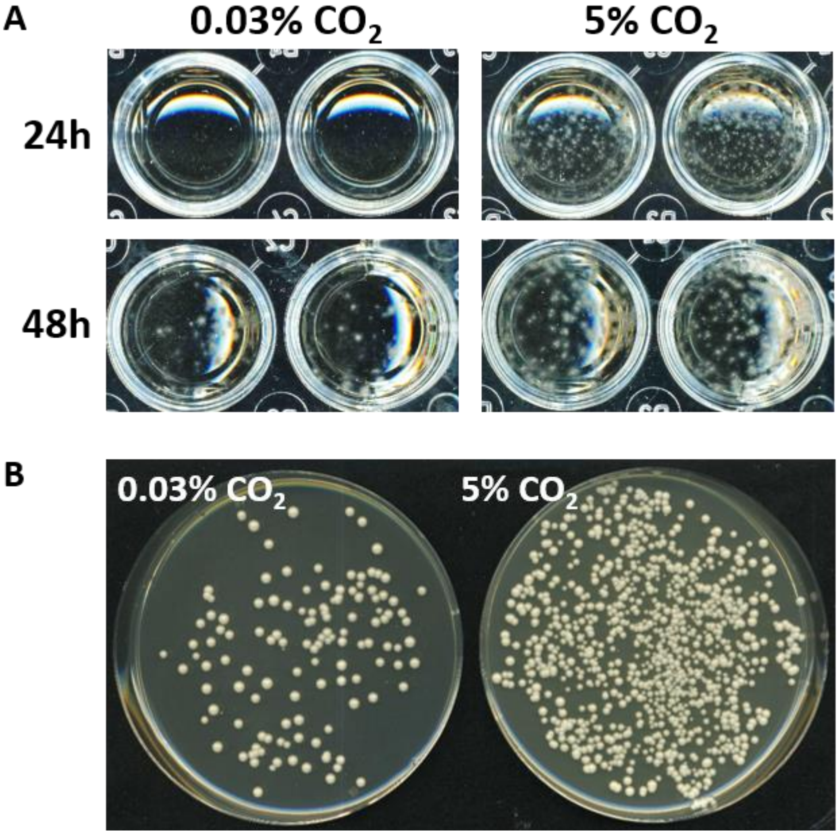

Supplementary Figure S4: Dispersion of *C. albicans* CAI4pSM2 cells from biofilms grown in 0.03% and 5% CO_2. (A) Wells containing RPMI-1640 media after biofilm growth, *C. albicans* cells can be seen as white clumps formed from hyphal cells. **(B)** Representative CFU plates from a 1:10 dilution of the spent RPMI-1640 media after 48h biofilm growth in both CO_2 conditions.

Supplementary Figure S5

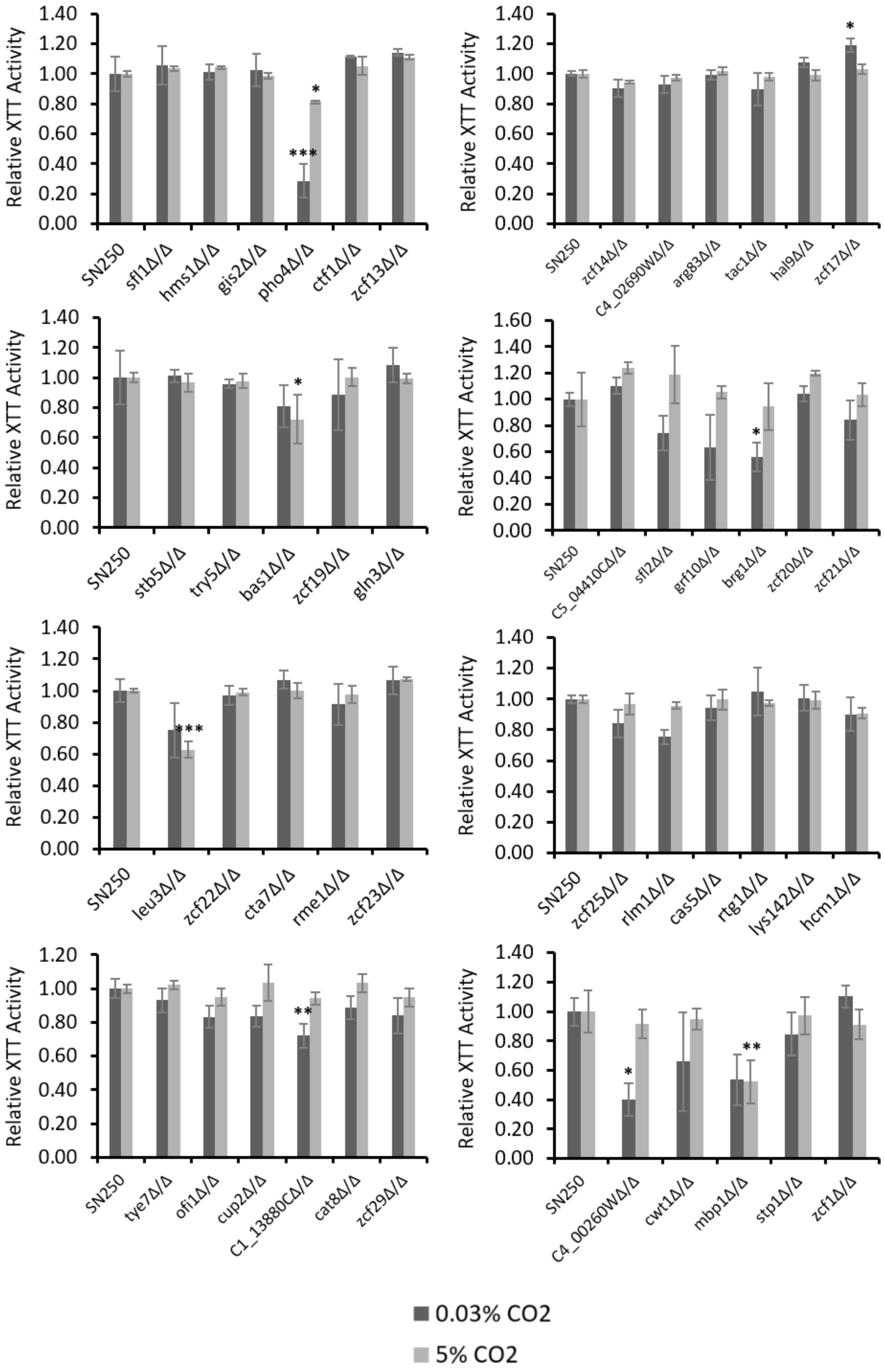

Supplementary Figure S5

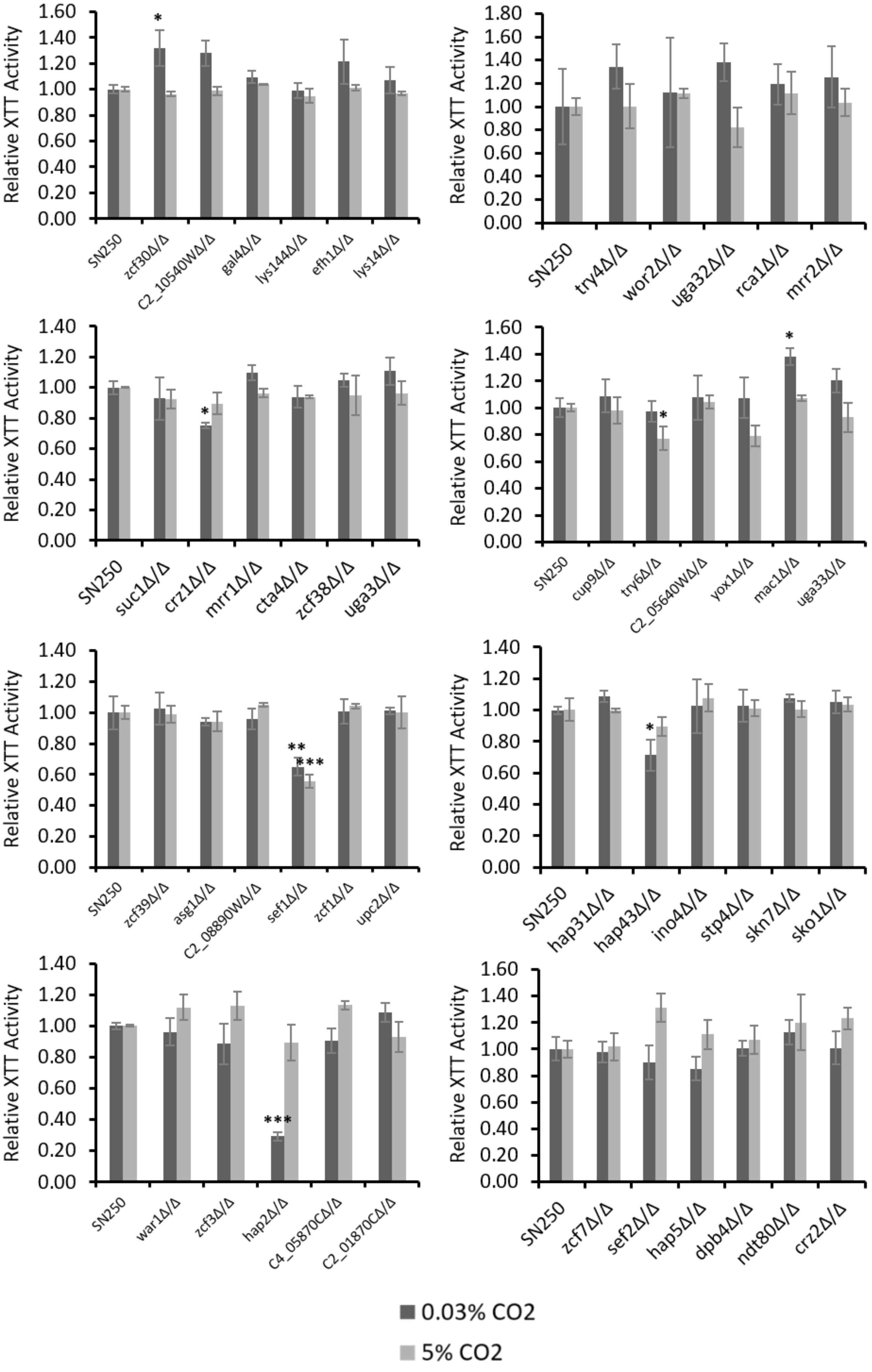

Supplementary Figure S5

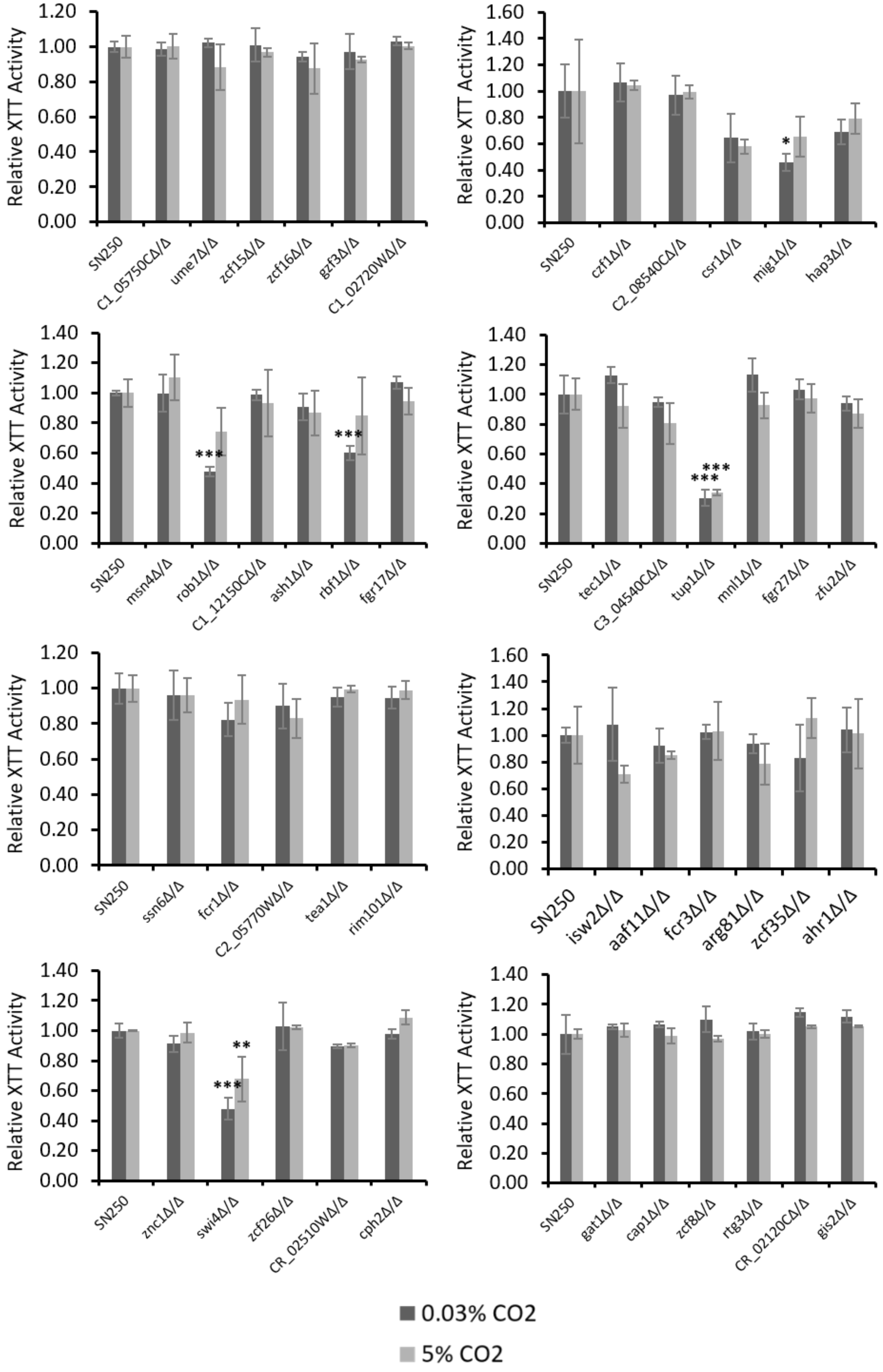

Supplementary Figure S5

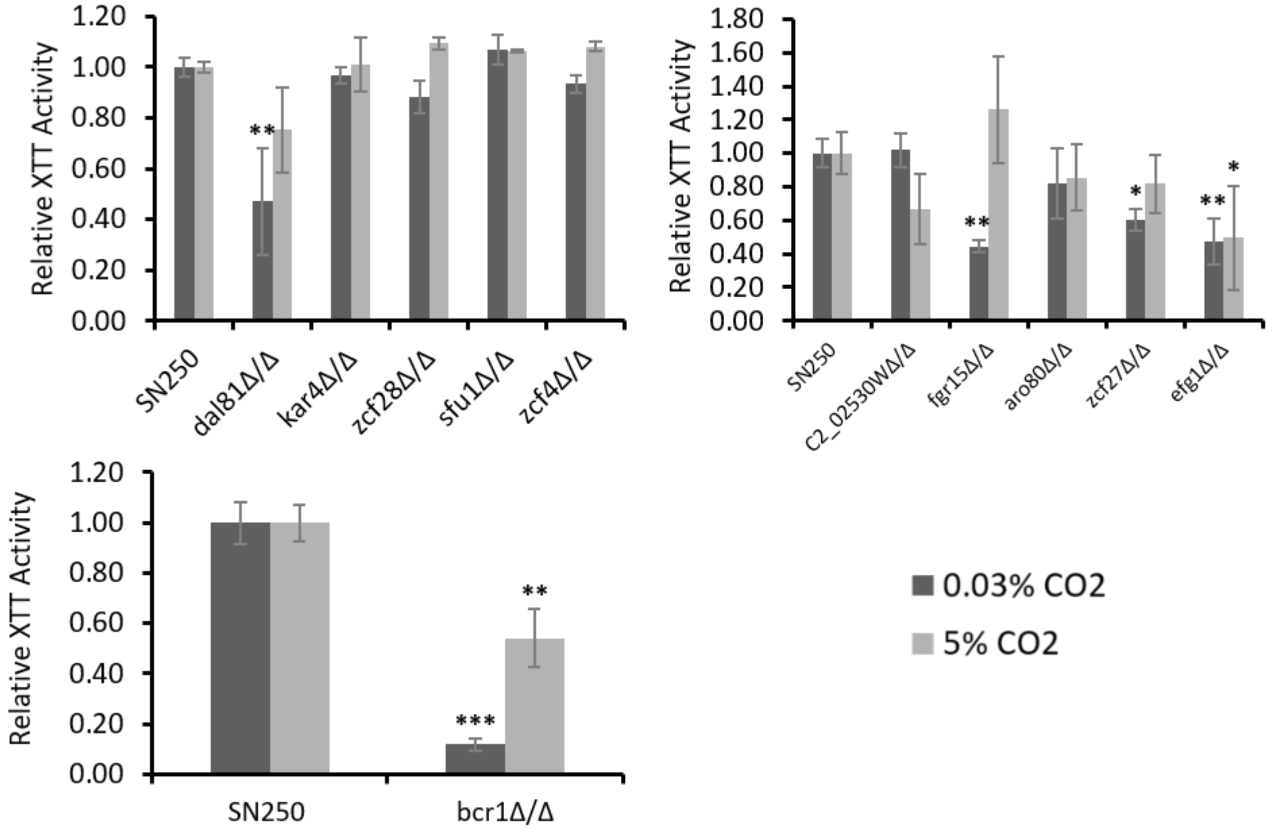

Supplementary Figure S5: Transcription Factor Knockout Screen of *C. albicans* biofilm-forming ability. Biofilms were seeded and grown before quantification via XTT assay. Graphs represent three biological replicates for each mutant. The XTT assay absorbances at 492nm for the 0.03% and 5% CO_2 biofilms have been normalised to the 0.03% and 5% CO_2 SN250 wild type controls respectively. This removes any day-to-day variation, thus allowing TFKO mutants grown on different days to be compared. One-way ANOVAs followed by Dunnett's Tests for multiple comparisons to a control were performed to compare the TFKO mutant biofilms to the SN250 wild type controls (for both 0.03% and 5% CO_2); *$p<0.05$, **$p<0.01$, ***$p<0.001$.

Supplementary Figure S6

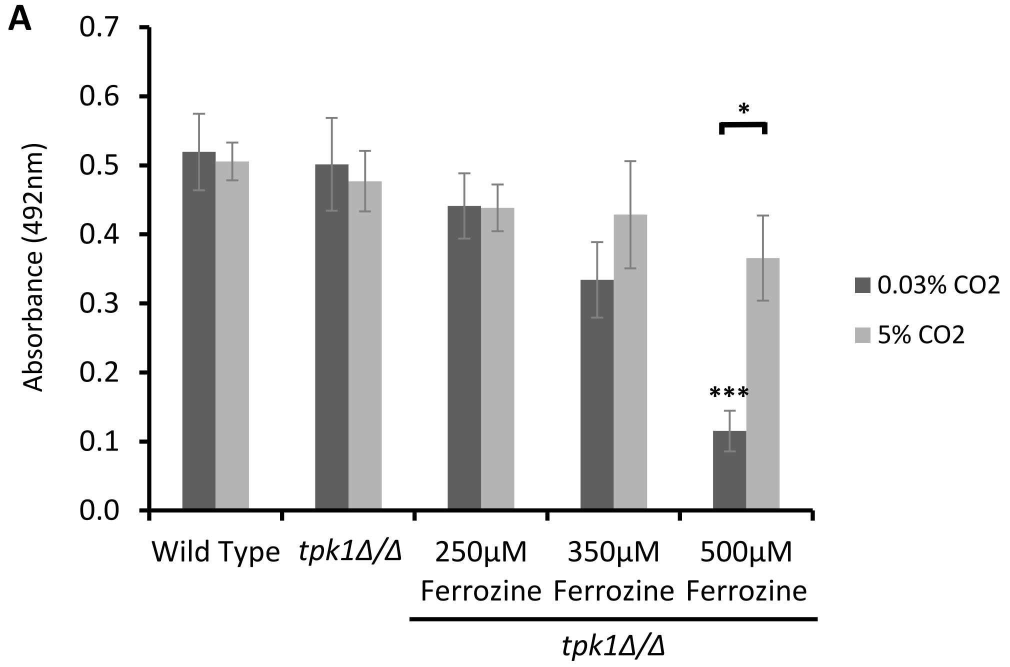

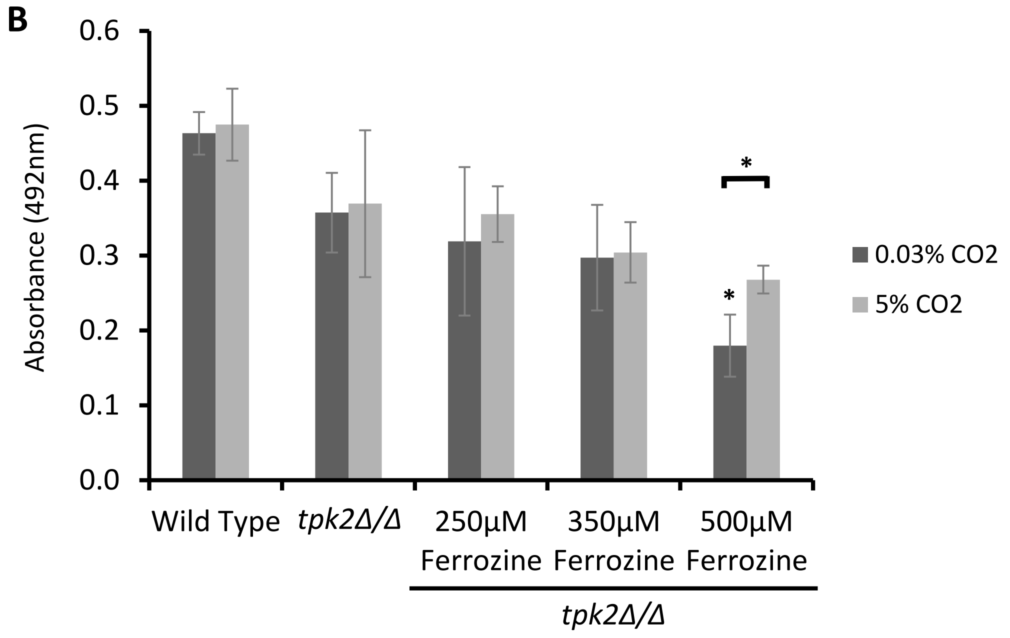

Supplementary Figure S6: The effect of high (5%) CO_2 on tolerance to iron starvation in *tpk1Δ/Δ* and *tpk2Δ/Δ* PKA mutants. Biofilms using **(A)** *tpk1Δ/Δ* and **(B)** *tpk2Δ/Δ* mutants were seeded and grown for 48h in the presence of the Fe^{2+} chelator Ferrozine before XTT quantification. Graphs represent two biological replicates each containing technical triplicates, error bars denote Standard Deviation. Two-way ANOVAs followed by Tukey tests for multiple comparisons were carried out: *p<0.05, **p<0.01, ***p<0.001. Stars directly above the bars indicate a significant difference to the untreated *tpk* deletion strain in the same CO_2 environment.

Supplementary Figure S7

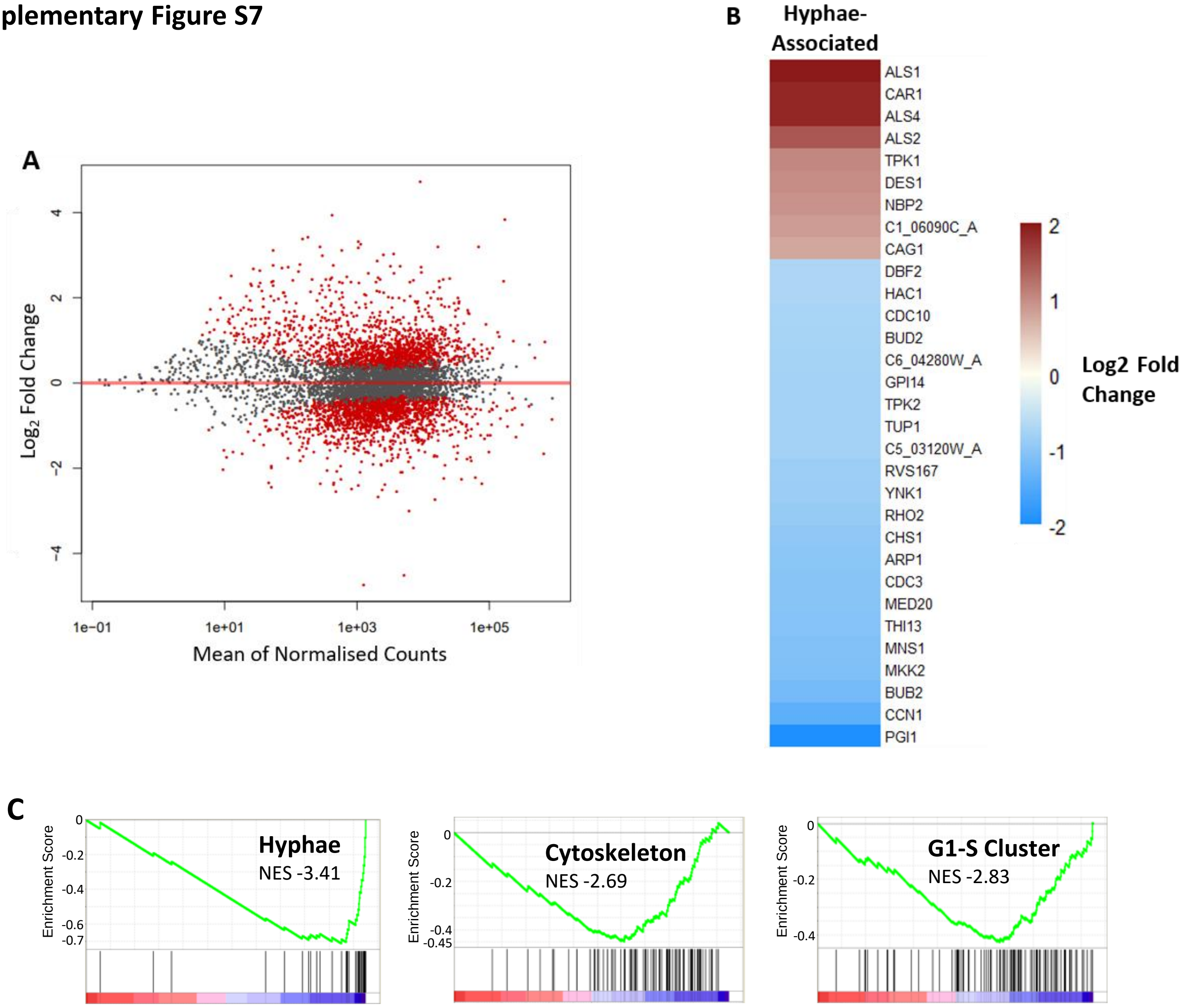

Supplementary Figure S7: Gene expression profiles of genes/gene sets downregulated in 5% CO_2 vs. 0.03% CO_2 *C. albicans* biofilms. (A) Volcano plot of log_2 fold changes (5% CO_2 vs. 0.03% CO_2 biofilms) against mean of normalised counts for each gene; red dots = significantly differentially expressed (q≤0.05) genes, grey dots = not significant. 2875 genes showed significant differential expression. Normalised counts are the number of reads a particular gene has, thus the higher the mean of normalised count, the higher the expression of that gene. **(B)** Heatmap of genes associated with hyphal growth as identified by GO term analysis. Colours saturate at log2 fold change of 2 and -2; *ALS1* actually has a log2 fold change of 3.77. **(C)** GSEA enrichment plots of the HYPHAE_FBS_37_UP gene set containing genes upregulated after 6h of exposure to FBS and 37°C (hyphal inducing conditions), the CYTOSKELETON_CEL gene set containing genes under the GO term 'cytoskeleton', and the G1-S CLUSTER gene set containing genes involved in the transition through the G1/S checkpoint. Vertical black lines represent individual genes in the ranked gene list from upregulated (left) to downregulated (right). NES = normalised enrichment score, negative NES indicates enrichment in the downregulated group of genes.

Supplementary Figure S8

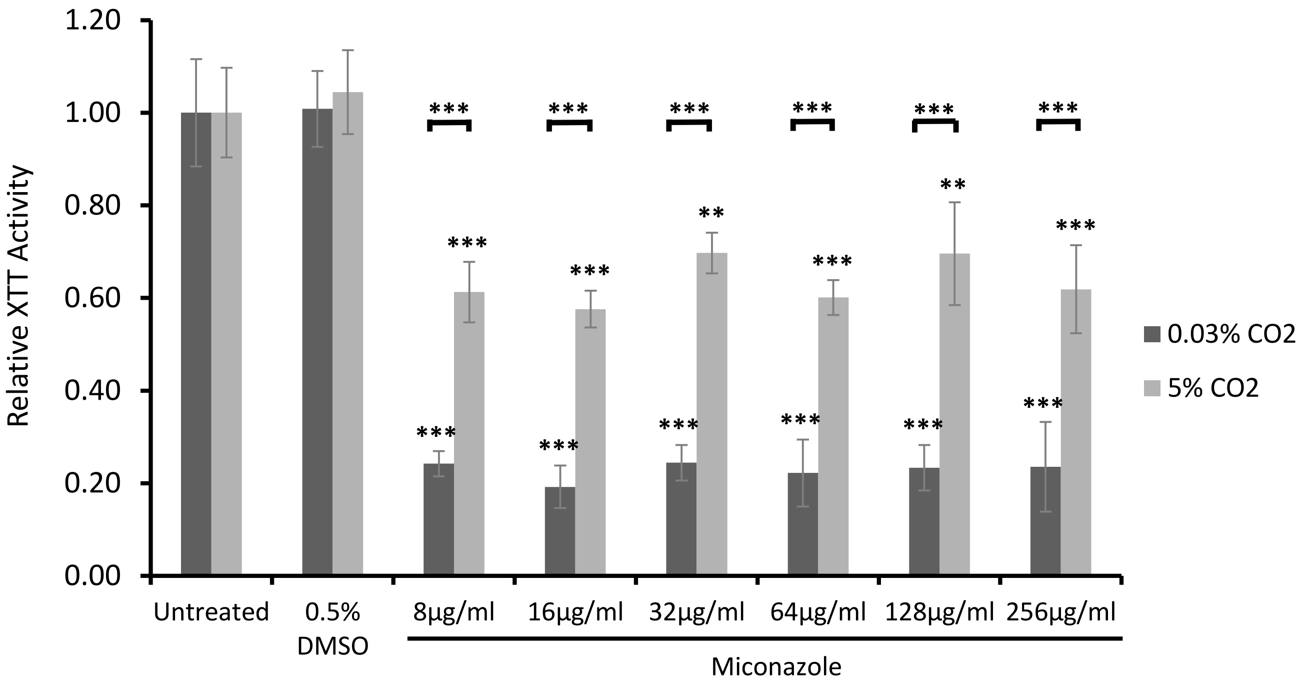

Supplementary Figure S8: Miconazole sensitivity of *C. albicans* biofilms grown in high (5%) CO_2. Biofilm growth assay of CAI4pSM2 in the presence of Miconazole. Biofilms were seeded and grown for 24 hours before antifungal addition, they were then grown for a further 24 hours before quantification using the XTT assay. The relative XTT activity is presented with the 0.03% CO_2 biofilms being normalised to the 0.03% CO_2 untreated control and the 5% CO_2 biofilms being normalised to the 5% CO_2 untreated control. This prevents the general higher growth of 5% CO_2 biofilms impacting the analysis. Two-way ANOVAs followed by Tukey tests for multiple comparisons were carried out: *p<0.05, **p<0.01, ***p<0.001. Stars directly above the bars indicate a significant difference to untreated in the same CO_2 environment.

Supplementary Figure S9

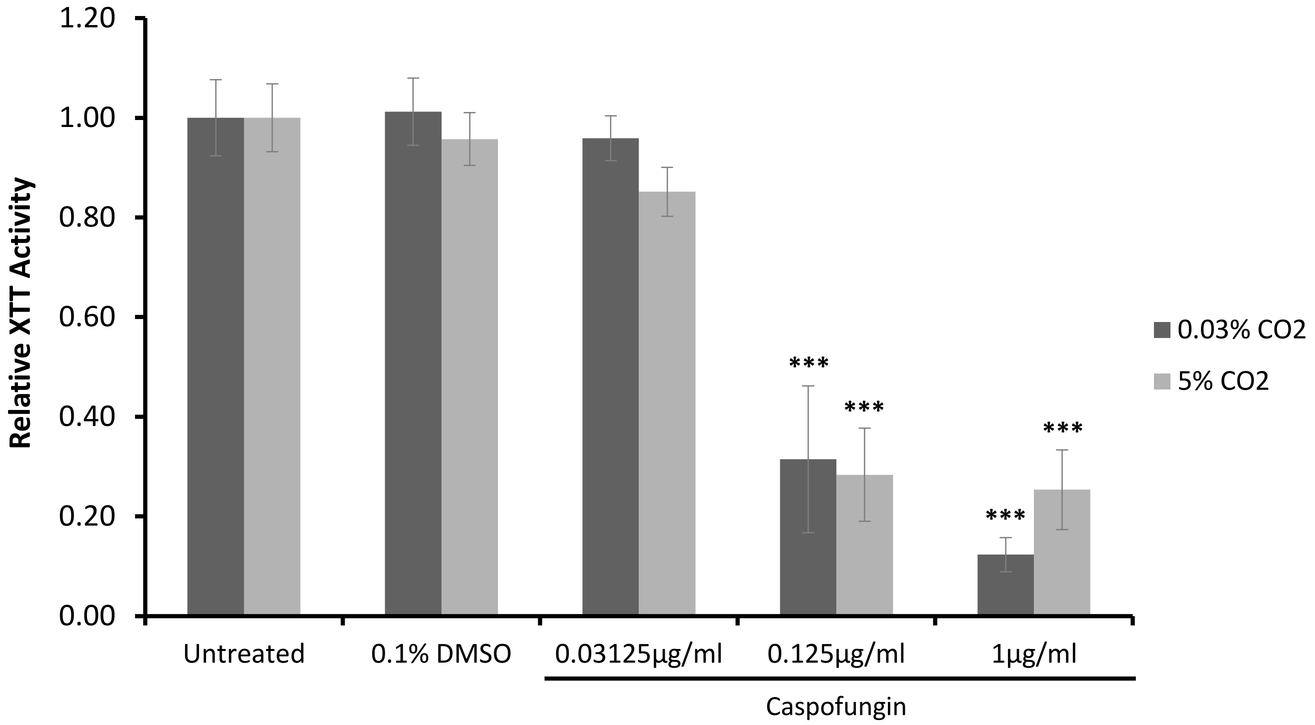

Supplementary Figure S9: Caspofungin sensitivity of *C. albicans* biofilms grown in high (5%) CO_2. Biofilm growth assay of CAI4pSM2 in the presence of Caspofungin. Biofilms were seeded and grown for 24 hours before antifungal addition, they were then grown for a further 24 hours before quantification using the XTT assay. The relative XTT activity is presented with the 0.03% CO_2 biofilms being normalised to the 0.03% CO_2 untreated control and the 5% CO_2 biofilms being normalised to the 5% CO_2 untreated control. This prevents the general higher growth of 5% CO_2 biofilms impacting the analysis. Two-way ANOVAs followed by Tukey tests for multiple comparisons were carried out: ***$p<0.001$. Stars directly above the bars indicate a significant difference to untreated in the same CO_2 environment.

Supplementary Figure S10

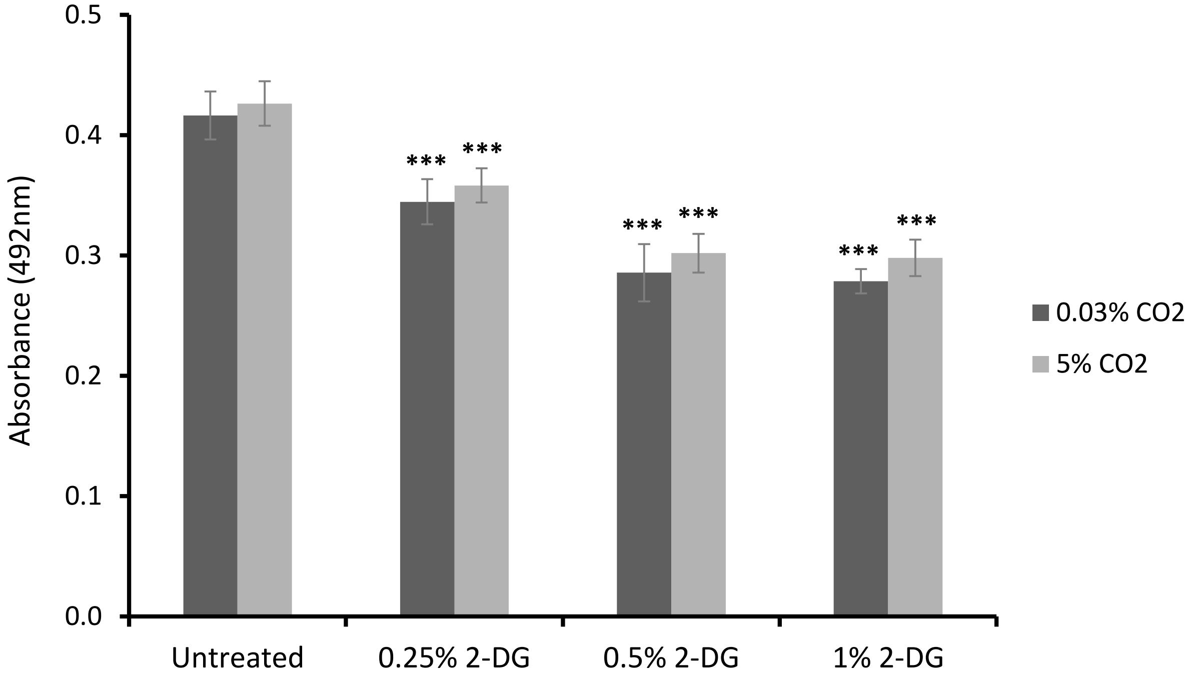

Supplementary Figure S10: Treatment of CAI4pSM2 *C. albicans* biofilms with 2-DG in 0.03% and 5% CO_2. Biofilms were seeded and grown for 48h in the presence of the glycolytic inhibitor 2-DG before XTT quantification. Control wells with no cells were set up as media controls to monitor for contamination as well as to ensure there was no reaction of the silicone squares with the XTT reagents. Graph represents three biological replicates each containing technical triplicates, error bars denote Standard Deviation. Two-way ANOVAs followed by Tukey tests for multiple comparisons were carried out: ***$p<0.001$. Stars directly above the bars indicate a significant difference to the untreated CAI4pSM2 in the same CO_2 environment.

Unprocessed western blot membrane images used within Figure 4C of this paper

Images below come from a single membrane cut and both pieces processed alongside each other on the same day.

Probed with anti-c-myc 9E10 antibody (Sigma: M4439)

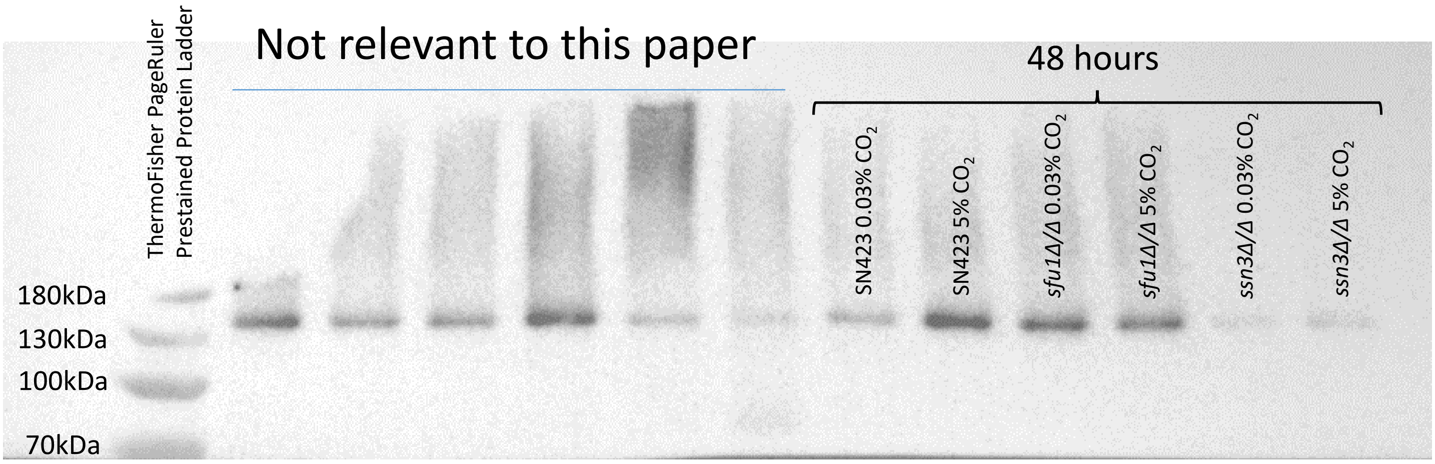

Probed with anti-PGK1 antibody (a gift from MF Tuite Lab, University of Kent)

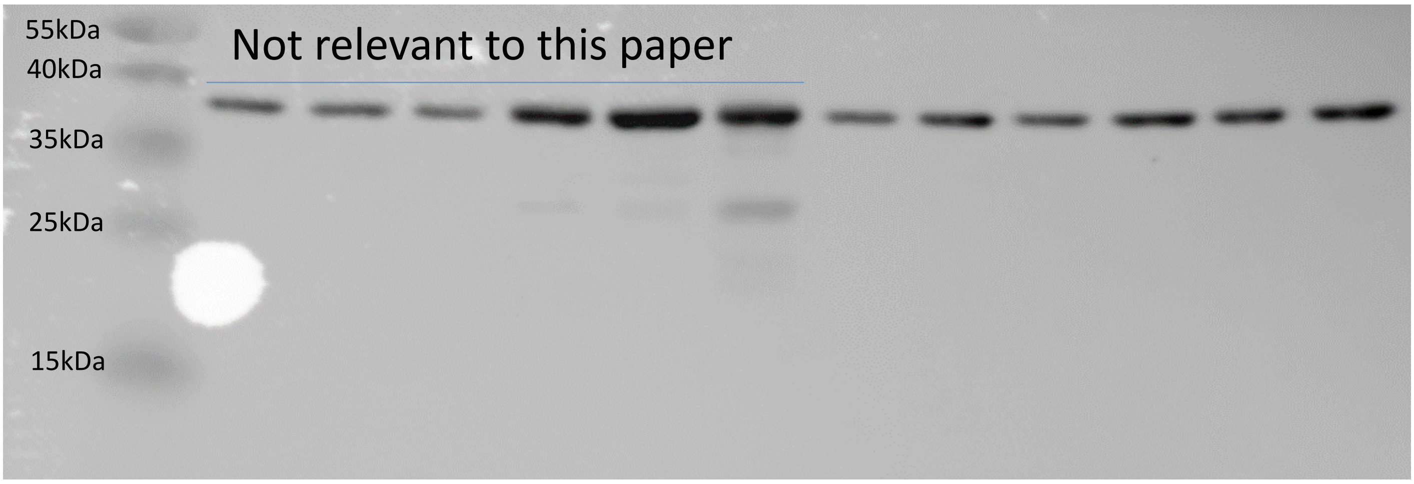

Supplement: Supplementary file 1 — Supplementary Information [file 41522_2021_238_MOESM1_ESM.pdf]
