# Supplementary material for: CO2 enhances the formation, nutrient scavenging and drug resistance properties of C. albicans biofilms
Source: NPJ Biofilms Microbiomes. 2021 Aug 12;7:67. doi: 10.1038/s41522-021-00238-z (PMC8361082; doi:10.1038/s41522-021-00238-z)
Supplement: Supplementary file 2 — Reporting Summary [file 41522_2021_238_MOESM2_ESM.pdf]

nature portfolio

Corresponding author(s): Campbell W. Gourlay

Last updated by author(s): Jul 13, 2021

Reporting Summary

Nature Portfolio wishes to improve the reproducibility of the work that we publish. This form provides structure for consistency and transparency in reporting. For further information on Nature Portfolio policies, see our Editorial Policies and the Editorial Policy Checklist.

Statistics

For all statistical analyses, confirm that the following items are present in the figure legend, table legend, main text, or Methods section.

n/a	Confirmed	
☐	☒	The exact sample size (*n*) for each experimental group/condition, given as a discrete number and unit of measurement
☐	☒	A statement on whether measurements were taken from distinct samples or whether the same sample was measured repeatedly
☐	☒	The statistical test(s) used AND whether they are one- or two-sided<br>*Only common tests should be described solely by name; describe more complex techniques in the Methods section.*
☒	☐	A description of all covariates tested
☐	☒	A description of any assumptions or corrections, such as tests of normality and adjustment for multiple comparisons
☐	☒	A full description of the statistical parameters including central tendency (e.g. means) or other basic estimates (e.g. regression coefficient) AND variation (e.g. standard deviation) or associated estimates of uncertainty (e.g. confidence intervals)
☐	☒	For null hypothesis testing, the test statistic (e.g. *F*, *t*, *r*) with confidence intervals, effect sizes, degrees of freedom and *P* value noted<br>*Give P values as exact values whenever suitable.*
☒	☐	For Bayesian analysis, information on the choice of priors and Markov chain Monte Carlo settings
☒	☐	For hierarchical and complex designs, identification of the appropriate level for tests and full reporting of outcomes
☒	☐	Estimates of effect sizes (e.g. Cohen's *d*, Pearson's *r*), indicating how they were calculated

Our web collection on statistics for biologists contains articles on many of the points above.

Software and code

Policy information about availability of computer code

Data collection	All data were collected using Microsoft excel
Data analysis	All data were analysed using Graphpad prism or R studio as stated within the materials and methods section of the paper

For manuscripts utilizing custom algorithms or software that are central to the research but not yet described in published literature, software must be made available to editors and reviewers. We strongly encourage code deposition in a community repository (e.g. GitHub). See the Nature Portfolio guidelines for submitting code & software for further information.

Data

Policy information about availability of data

All manuscripts must include a data availability statement. This statement should provide the following information, where applicable:

- Accession codes, unique identifiers, or web links for publicly available datasets
- A description of any restrictions on data availability
- For clinical datasets or third party data, please ensure that the statement adheres to our policy

RNA Sequencing data that support the findings of this study have been deposited in the Gene Expression Omnibus (GEO) repository with the accession code GSE172004. All other data that support the findings of this study are available from the corresponding author upon reasonable request.

Field-specific reporting

Please select the one below that is the best fit for your research. If you are not sure, read the appropriate sections before making your selection.

☒ Life sciences ☐ Behavioural & social sciences ☐ Ecological, evolutionary & environmental sciences

For a reference copy of the document with all sections, see nature.com/documents/nr-reporting-summary-flat.pdf

Life sciences study design

All studies must disclose on these points even when the disclosure is negative.

Sample size	All experiments were carried out in biological replicates of at least n=3 and in all cases the replicate number is stated. Statistically significant differences between dependent variables were assessed with either a paired two-tail t-test or, more often, with a two-way ANOVA followed by a Tukey test for multiple comparisons. All statistical analyses were carried out using GraphPad Prism, version 9.0.2.
Data exclusions	No data were excluded from the analyses in this paper
Replication	All findings in this article were reproducible as assessed by biological replication and appropriate statistical analyses
Randomization	All findings are based on average readouts of a population of cells and assume equal variance.
Blinding	Blinding was carried out within this study, all experiments contained appropriate controls for comparison as a means to determine the effect of single parameter change within steady state conditions.

Reporting for specific materials, systems and methods

We require information from authors about some types of materials, experimental systems and methods used in many studies. Here, indicate whether each material, system or method listed is relevant to your study. If you are not sure if a list item applies to your research, read the appropriate section before selecting a response.

Materials & experimental systems

n/a	Involved in the study	
☐	☒	Antibodies
☒	☐	Eukaryotic cell lines
☒	☐	Palaeontology and archaeology
☒	☐	Animals and other organisms
☒	☐	Human research participants
☒	☐	Clinical data
☒	☐	Dual use research of concern

Methods

n/a	Involved in the study	
☒	☐	ChIP-seq
☒	☐	Flow cytometry
☒	☐	MRI-based neuroimaging

Antibodies

Antibodies used	9E10 anti-c-myc antibody (Sigma-Aldrich, M4439) for Myc-tagged Sef1. Immunoblots were also probed with an anti-PGK1 antibody (gift from the M. F. Tuite lab, University of Kent)
Validation	Validation was achieved by comparison to untagged strains lacking the c-myc antigen (not found in yeast) and by expected size increase of tagged protein (M4439), validation is also provided on the supplier website by recognition of purified antigen (https://www.sigmaaldrich.com/GB/en/product/sigma/sab4301136?context=product). The PGK antibody has been validated by the Tuite lab in a number of publications eg https://www.ncbi.nlm.nih.gov/pmc/articles/PMC3399540/
